# Chemical and Conformational Diversity of Modified Nucleosides Affects tRNA Structure and Function

**DOI:** 10.3390/biom7010029

**Published:** 2017-03-16

**Authors:** Ville Y. P. Väre, Emily R. Eruysal, Amithi Narendran, Kathryn L. Sarachan, Paul F. Agris

**Affiliations:** The RNA Institute, Departments of Biological Sciences and Chemistry, University at Albany, State University of New York, Albany, NY 12222, USA; eeruysal@albany.edu (E.R.E.); anarendran@albany.edu (A.N.); KSarachan@albany.edu (K.L.S.)

**Keywords:** tRNA modifications, physicochemical properties of RNA modifications, chemical biology of RNA modifications, RNA modified nucleoside contributions to function

## Abstract

RNAs are central to all gene expression through the control of protein synthesis. Four major nucleosides, adenosine, guanosine, cytidine and uridine, compose RNAs and provide sequence variation, but are limited in contributions to structural variation as well as distinct chemical properties. The ability of RNAs to play multiple roles in cellular metabolism is made possible by extensive variation in length, conformational dynamics, and the over 100 post-transcriptional modifications. There are several reviews of the biochemical pathways leading to RNA modification, but the physicochemical nature of modified nucleosides and how they facilitate RNA function is of keen interest, particularly with regard to the contributions of modified nucleosides. Transfer RNAs (tRNAs) are the most extensively modified RNAs. The diversity of modifications provide versatility to the chemical and structural environments. The added chemistry, conformation and dynamics of modified nucleosides occurring at the termini of stems in tRNA’s cloverleaf secondary structure affect the global three-dimensional conformation, produce unique recognition determinants for macromolecules to recognize tRNAs, and affect the accurate and efficient decoding ability of tRNAs. This review will discuss the impact of specific chemical moieties on the structure, stability, electrochemical properties, and function of tRNAs.

## 1. Introduction

The central role of RNA in cellular function has been well established through numerous studies. Not only does RNA translate the genetic code decoding it into protein, but it also has various catalytic and regulatory functions yet to be completely elucidated [1,2,3,4]. RNA versatility and the complex roles which it plays in cellular life and function are possible through its extensive variation in length from microRNAs and transfer RNAs (tRNAs) of less than 100 nucleosides in length, to long non-coding RNAs (lncRNAs), messenger RNAs (mRNAs) and ribosomal RNAs (rRNAs) of 1000s of nucleosides in length. Its six backbone torsion angles and glycosidic bond between base and ribose provide conformational dynamics not afforded by the peptide bond of proteins and more flexibility than the DNA backbone. RNA has over 100 different post-transcriptional modifications [5,6]. The post-transcriptional modifications are enzymatically inserted at site-specific locations, for instance the methylation of uridine to 5-methyluridine or ribothymidine is almost invariant at position 54 in all tRNAs. Some of the modifications are quite common, such as 2’-*O*-methylation and pseudouridylation. Others are found uniquely in one RNA species such as the complex tricyclic modification of G to wyosine (9*H*-imidazo[1,2-*a*]purin-9-one,3,4-dihydro-4,6-dimethyl-3-*b*-d-ribofuranosyl-) found at position 37 in tRNA^Phe^ of eucaryotes. The chemical, dynamic and structural properties of the modified nucleosides far surpass those of the individual characteristics provided by the four major nucleosides: the purine (Pu) ribonucleosides, adenosine (A) and guanosine (G), and the pyrimidine (Py) ribonucleosides, cytidine (C) and uridine (U) [2]. The chemical diversity of modifications is similar to that of amino acid side chains and can be as simple as methylations or single atom substitutions of sulfur for oxygen to as complex as the addition of amino acids and formation of a third ring to the purine nucleobase [7]. Even modifications as simple as methyl groups can exhibit distinguishable dynamic motions that depend on their location within the RNA structure, for instance the methyl groups of yeast tRNA^Phe^ [8]. The prodigious chemical variation provided by the numerous modifications hitherto discovered allow RNA to perform numerous cellular functions [2].

**Figure 1:**
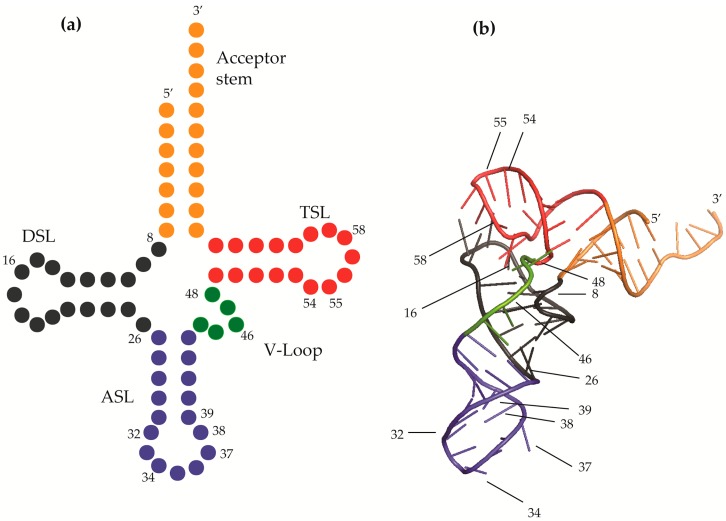
Transfer RNA (tRNA) secondary and tertiary structure. Yeast initiator tRNA cloverleaf structure (Left) and three-dimensional L-shape structure (Right) are shown with selected modification sites labeled. The colors correspond to the acceptor stem (orange), D stem and loop (DSL) domain (gray), T stem and loop (TSL) domain (red), anticodon stem and loop (ASL) domain (blue), and variable loop (V-Loop) domain (green). Three-dimensional figure adopted from Protein Data Bank (PDB) file 1YFG [16]. Weakly binding Mg^2+^ ions are key to the folding of tRNA, as they are to all RNAs. They are shown in Figure 3. One Mg^2+^ binding site is in the loop of the ASL and often found in the vicinity of ASL modifications.

Although the existence of modified nucleosides has been known for over six decades, much of how modified nucleosides alter the structure, function, and properties of RNAs is still unknown. More than a hundred modified nucleosides can be found in all domains of life in mRNA, rRNA, and other RNAs such as small nucleolar RNA (snoRNA) and small nuclear RNA (snRNA). Still, the greatest number and variety of modifications can be found in tRNAs (Figure 1) [1].

**Figure 2:**
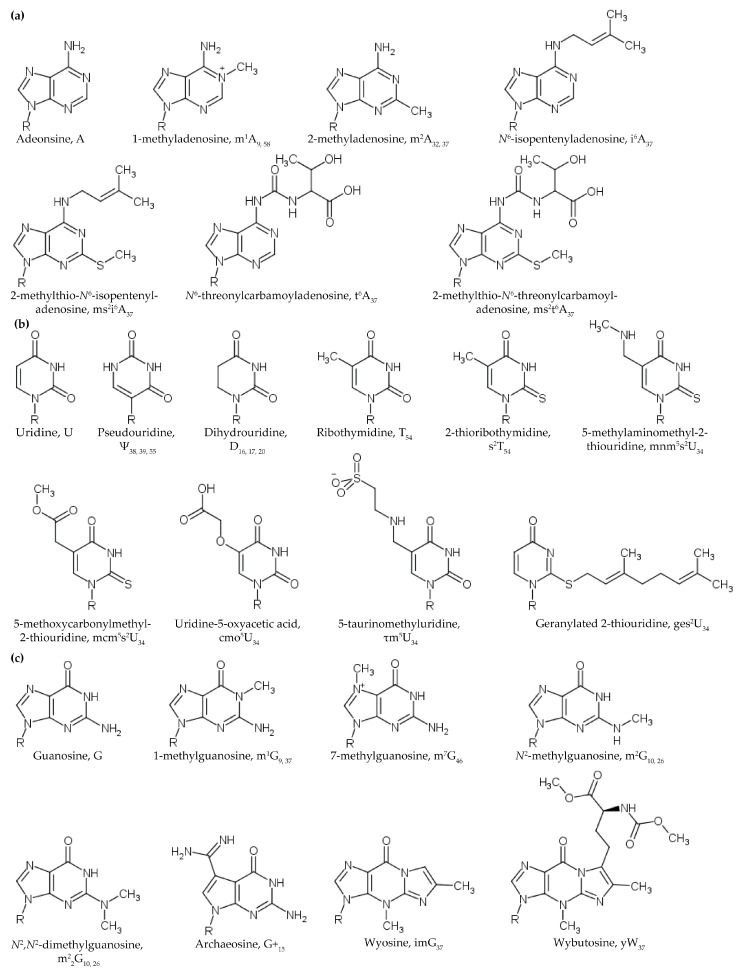

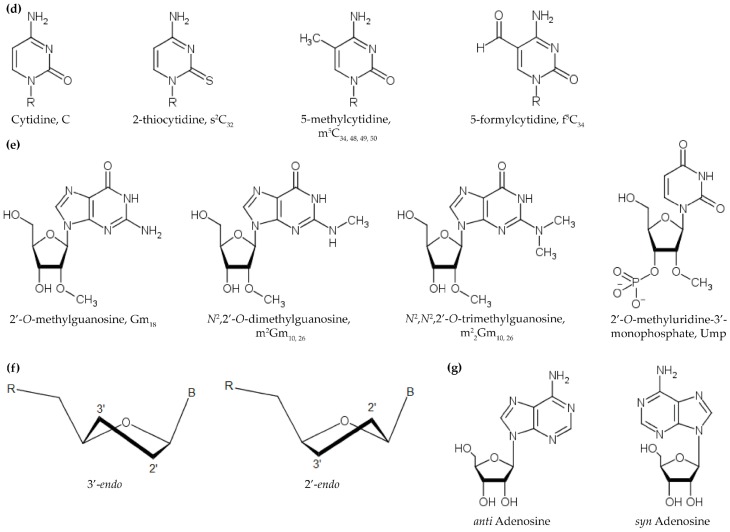
Nucleoside Modifications and Conformations. Some 90 modified nucleosides appear in tRNAs. The modifications, nucleobase and ribose conformations addressed in this review are (**a**) Adenosine and its modifications (R = ribose); (**b**) Uridine and its modifications (R = ribose); (**c**) Guanosine and its modifications (R = ribose); (**d**) Cytidine and its modifications (R = ribose); (**e**) 2’-*O*-methylated nucleosides; (**f**) C3’-*endo* versus C2’-*endo* (R = OH or phosphate; B = nucleobase); (**g**) *anti* adenosine versus *syn* adenosine.

Modifications of the nucleobases and of the 2’-OH of the ribose are crucial for various biological processes (Figure 2). While the biologically important role of RNA modifications has been extensively studied, the physicochemical nature of the RNA modifications is also of interest because these properties enable or facilitate RNA function. Reviews specifying the chemical moieties found at various positions of tRNA can be found elsewhere [5,9,10,11,12,13,14,15]. Here, we will focus on the impact of specific modified nucleoside chemistries which add to the chemistry of tRNAs and alter their structure and stability.

In general, modified nucleosides impact the overall structure and local chemistry of tRNAs, the ability of macromolecules to recognize tRNAs, or the decoding ability of tRNAs [2]. Specific modifications commonly found at the junction of the stems of the cloverleaf structure (Figure 1), referred to as the core, elbow or hinge in the three-dimensional structure of most tRNAs, are often associated with functional folding, allowing tRNAs to fold into the common L-shape form critical to ribosomal protein synthesis [1]. Similarly, common features found in several tRNAs are often used by proteins and other macromolecules as recognition determinants to interact with specific tRNA species, or tRNAs in general [1,17]. The properties and modified nucleosides of the tRNA core are often used as chemical and structural determinants, such as in the case of tRNA-dependent gene regulation in Gram-positive bacteria, the T-box mechanism [18].

Certain modifications are only found at very specific locations, whereas others can be found in a number of locations common to many tRNA species (Figure 1) [19]. For example, the purine at position 37 of a number of tRNAs is modified with a bulky moiety not found at other positions [20]. Similarly, tRNAs with a uridine at wobble position-34 read A-ending codons. The wobble position uridines are often modified with a methylene carbon directly bonded to the C5 atom of the uracil nucleobase (xm^5^U) with a variety of further derivations. The xm^5^U modifications are indicative of a specificity required of tRNA for the accurate decoding of mRNA [1,2]. In contrast, pseudouridine (Ψ) is found at multiple positions such as 38, 39, and 55 among others. The overall structure of tRNA is folded correctly in part due to certain modifications, particularly methylations, found at the core. The three-dimensional L-shape is maintained with the help of modified nucleosides that drive the structure into forming strong, favorable interactions between the D- and T-arms. Furthermore, the local chemical environment can be either stabilized or destabilized by a single modified nucleoside to allow for greater rigidity or flexibility, respectively, through stacking and hydrogen bonding capabilities. Complex modifications at the wobble position and position 37 have both structural as well as functional roles imperative to decoding. Here, we discuss and cite examples of how the chemical and dynamic nature of the modified nucleosides affects their ability to elicit functionally important properties of tRNA, cloverleaf secondary structure, L-shaped three-dimensional structure, anticodon conformation and dynamics.

## 2. Modifications Responsible for tRNA Structure

The tertiary structure of tRNA is maintained by stable interactions among the conserved and semi-conserved residues of the tRNA molecule. The L-shaped tRNA tertiary structure has three distinct regions: an acceptor stem at the 3’-terminus at which aminoacylation takes place; an anticodon stem and loop (ASL) which decodes mRNA codons at the ribosome; and the tRNA ‘core’, which is composed of the D-loop named after the conserved dihydrouridine within the loop of the D-stem and loop (DSL) domain, the V-loop (VL) or variable region named for its variation in length, also known as the extra loop, and the TΨC loop, named after the conserved ribothymidine–pseudouridine–cytidine sequence found in the loop of the T-stem and loop (TSL) domain (Figure 1). The three-dimensional core of the L-shaped three-dimensional structure consists of several stacked layers of tertiary base pairs and base triples [21]. The core is constituted, in general, from the cloverleaf secondary structure by the interlocking of the D- and T-loops forming the hinge of the L-shaped tRNA fold [22,23,24]. Stacked base interactions occur between adjacent bases and long range tertiary hydrogen bonds between the bases and the sugar phosphate backbone [25]. Due to differences in the tertiary interactions, sequence variations, and length of D- and variable loops, there can be important conformational differences in tRNAs. Individual bases involved in the stacking and hydrogen bonding of the core vary for each tRNA, however, the sequence variations among tRNA species occur in such a way as to preserve the global architecture of the structure required of protein synthesis [26,27].

Conformational changes play an important role in the specific functions of tRNA and they are also important to the dynamics of its core structure [22]. Efficient translation on the ribosome is dependent on preservation of the general structure and conformational dynamics of the core among all tRNA species. Yet, the ribosome-accepted structure is fashioned from tRNAs with sequence variation. Aminoacylation of tRNA, while governed solely by the nucleosides in the acceptor stem for some tRNAs and primarily by the nucleosides in the acceptor stem and ASL for most tRNAs, it is also dependent on the structural features and nucleosides of the core [28]. The aminoacyl-tRNA synthetase determinants for recognition of *Escherichia coli* tRNA^Arg^ and yeast tRNA^Phe^ are located at the junction of the loops for the DSL and TSL [29,30]. Therefore, the architecture and dynamics of the three-dimensional core of tRNA are a contributing factor to the specificity of aminoacylation.

### 2.1. tRNA Junction

#### 2.1.1. Position 9 and 10 (A_9_, G_9_ and G_10_)

Nucleoside positions 9 and 10 of the tRNA cloverleaf two-dimensional structure are important to the RNA’s functional folding. Especially evident are their contributions to the folding of mitochondrial (mt) tRNAs with truncated DSLs and/or TSLs. The methylations resulting in *N*^1^-methylguanosine, m^1^G_9_, and *N*^1^-methyladenosine, m^1^A_9_, have been implicated in the correct folding of mitochondrial tRNA [31,32,33,34]. Lack of the m^1^G_9_ and m^1^A_9_ modifications through mutation of one of the cytoplasmic orthologs of the methyltransferase (TRMT10A) has been associated with microcephaly, intellectual disability, and short stature [35]. Modification of A_9_ to m^1^A_9_ in the human mitochondrial tRNA^Lys^ (mt tRNA^Lys^) disrupts formation of an intra-stem Watson–Crick base pair. The methylation to m^1^A_9_ is sufficient to shift the equilibrium from a stable alternative, non-canonical extended hairpin structure to the more favored, and functional cloverleaf secondary structure [31,36,37]. The extended hairpin structure although predominating in a dynamic equilibrium with the cloverleaf structure, was not a substrate for aminoacylation by lysyl-tRNA synthetase [38], nor functional on the ribosome. The m^1^A_9_ was thought to be an early maturation event occurring after maturation of the 5’ terminus of mt tRNA^Lys^ by RNAse P [39]. The presence of the methyl group of m^1^A_9_ in mt tRNA^Lys^ stabilizes the cloverleaf structure by 3–4 kJ/mol and is dependent on the Mg^2+^ concentration [40]. Such structural plasticity is a common characteristic in animal mitochondrial tRNAs including mt tRNA^Leu^_UUU_ and mt tRNA^Asp^ where the cloverleaf structure is attained often after certain bases have been modified [41], and with Mg^2+^ coordination [42].

By example, the mitochondrial tRNAs of the nematode *Ascaris suum* lack either the TSL or the DSL found in the tRNAs of most organisms and are characterized by the presence of m^1^A_9_. tRNAs without this modification had reduced aminoacylation and lower EF-Tu (elongation factor thermos unstable) binding activities compared to native tRNAs [32]. Aminoacylation and tertiary structure were nearly restored to normal by the presence of a single methyl group, indicating the importance m^1^A_9_ in the absence of the TSL in mitochondrial tRNAs in nematodes, and other organisms. Comparable to m^1^A_9_ in mt tRNA, *N*^1^-methylguanosine (m^1^G) disrupts canonical base pairing in mt and cytoplasmic tRNAs by virtue of the methyl-group blocking the Watson–Crick face of the nucleoside, thus disturbing secondary structures formation [43].

#### 2.1.2. G10: 2-Methylguanosines

**Figure 3:**
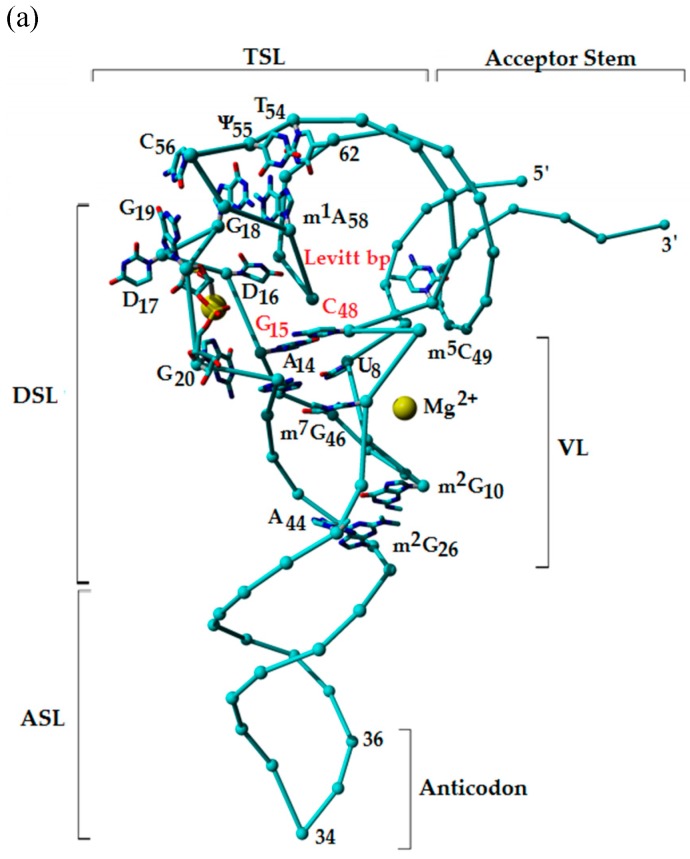

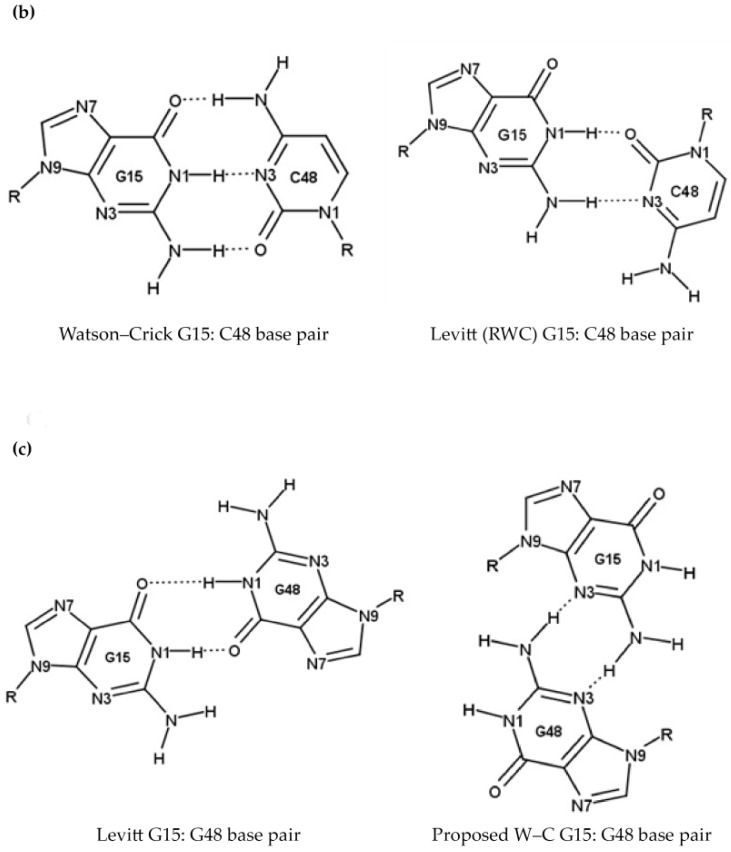
tRNA core structure and Levitt base pair model. (**a**) A three-dimensional model of the tRNA^Phe^ tertiary structure, adapted from PDB File 1EHZ [46]. Nucleoside 15 in the variable loop forms a non-canonical base pair known as the ‘Levitt pair’ with the nucleoside 48 in the loop of the D stem and loop. The Levitt base pairing between nucleosides G_15_ and C_48_ is shown along with the U_8_:A_14_ stacked bases below the G○C base pair. The open circle denotes a non-canonical base pair. Magnesium ions, found at sites at which modifications also occur, are shown as dark yellow. Other modified nucleosides discussed as important to the tRNA core stability and functions are listed. VL: variable loop. (**b**) The canonical Watson–Crick (W–C) base pairing between G_15_○C_48_ pairs and the Levitt base pair with the reverse W–C (RWC) geometry is formed between two antiparallel strands, by rotation of one base to the *syn* orientation. (**c**) The G_15_○G_48_ tertiary H-bonding in *Escherichia coli* tRNA^Cys^. The *trans* orientation of the glycosidic bonds in G_15_ and G_48_ (left) allows a W–C base pairing like the G_15_○C_48_ in yeast tRNA^Phe^. The proposed base pairing on the right is stabilized by hydrogen bonding between the exocyclic N-2 with the ring N3 of the base.

During tRNA synthesis, processing and modification, it is important that the secondary and tertiary base-pairings which yield the functional folding of tRNA occur and be maintained afterwards. For functional folding to be achieved, the four major stems terminate at a junction within the cloverleaf secondary structure leaving an open core (Figure 1 and Figure 3a). Convergence of the stems produce an internal loop of the molecule. Methylations of nucleosides at the junctions of the accepting stem and DSL, and the DSL and ASL are excellent examples of stem interruptions leading to the internal loop which is the core of tRNAs. The stem interruptions by 2-methylated guanosines at position 10 at the junction of the acceptor stem and the stem of the DSL, and at position 26 between the stem of the DSL and that of the ASL facilitate the secondary and tertiary folding of tRNAs (Figure 1 and Figure 3). This family of structurally related nucleosides, *N*^2^-methylguanosine (m^2^G), *N*^2^,*N*^2^-dimethylguanosine (m^2^_2_G), and *N*^2^,*N*^2^,2′-*O*-trimethylguanosine (m^2^_2_Gm), are conserved at positions 10 and 26 and control the L-fold in the tertiary tRNA structure in all three domains of life [44]. The methyl groups, located on the Watson–Crick face of the nucleobase, negate canonical base pairing. The duplexes terminate at the modifications in bacteria, eukaryotes, and archaea tRNAs. For example, in the *Stetteria hydrogenophila* archaeal tRNAs, the modifications at the terminations of the duplexes play a crucial role in stabilizing tRNA conformation of the archaeal thermophiles—organisms thriving at inhospitably high temperatures [45]. However, experimental substitution of the methylated guanosines has demonstrated that the *N^2^*-methylguanosines play an additional role to that of terminating tRNA stems at the junction of the cloverleaf structure.

Studies using base substitutions have shown the importance of interactions of certain bases with proteins. Since an inosine–cytosine (I●C) base pair resembles a G●C base pair structurally, substitutions of G with inosine have been used to assess the role of guanosines in maintaining the helical structure. (The filled circle, ●, designates a canonical or similar base pair.) Early studies using inosine-substituted tRNA by recombinant RNA technology, identified the 2-amino group in guanosines at positions 2, 3 and 10 as important structural determinants of tRNA for protein recognition. Inosine substitutions at positions 2, 3 and 10 decreased in vitro aminoacylation of *E. coli* tRNA^Gln^ by glutaminyl-tRNA synthetase (GlnRS), with a 300-fold decrease in the specificity constant (*k_cat_/K_m_*) [47]. These studies reveal G_10_ as a key structural identity element for GlnRS recognition and demonstrated the importance of hydrogen bonding between the 2-amino groups of the guanosine with the synthetase amino acids. The highly conserved G_10_ in *E. coli* tRNAs [48] and the G_10_ interaction in the tRNA^Asp^–AspRS complex further suggest the importance of the methyl group and its effect on structure and dynamics as features recognized by the cognate aminoacyl-tRNA synthetases.

#### 2.1.3. Position 26 (G26)

Without going into detail, folding of the tRNA cloverleaf secondary structure into the L-shaped tertiary structure is a general architectural feature of tRNA necessary for aminoacylation and translation on the ribosome. These general structural features of tRNAs have also been shown to be a necessary global recognition element for modifying enzymes, such as the methyltransferases producing m^2^_2_G_26_ in yeast tRNA^Asp^ and tRNA^Phe^ [22,49]. Although m^2^_2_G_26_ in between the D-stem and the ASL is present in more than 80% eukaryotic tRNA containing a guanosine at position 26, many eukaryotic tRNAs have unmodified G_26_, e.g., yeast tRNA^Asp^. Studies using an in vitro modified dimethylguanosine in *Xenopus laevis* oocytes have identified the two consensus base pairs preceding G_26_ in the D-stem as a prerequisite for the *N^2^*,*N^2^*-dimethyl modification of G_26_, forming *N^2^*,*N^2^*-dimethylguanosine [49]. Furthermore, the global structure of the tRNA was important for protein recognition of nuclear encoded tRNA including the tRNA m^2^_2_G_26_ methyltransferase [49].

A decrease in tRNA synthesis affected by rapamycin correlates with an increase in m^2^_2_G_26_ modification; the correlation is conserved from yeast to humans [50]. As with many methyltransferases, the enzymatic synthesis of m^2^_2_G_26_ is sometimes outperformed by the rate of transcription. The m^2^_2_G_26_ modification is important for tRNA to translate the genetic code. The m^2^_2_G_26_ and m^2^G, unlike other methylations in eukaryotes and archaeal organisms catalyzed by the *S. cerevisiae* N2,N2-dimethylguanosine methyltransferase Trm1, are thought to prevent Watson–Crick base pairing and to stabilize the three-dimensional core (reviewed in [51]).

#### 2.1.4. Position 48 and 49 (m^5^C_48_ and m^5^C_49_)

The 5-methylcytosine (m^5^C) modification, although present in both tRNAs and ribosomal RNA in eukaryotes and archaea, is absent in *E. coli* tRNAs but is found in the ribosomal RNA of *E. coli* [27]. Plant mitochondria and chloroplast tRNAs are devoid of m^5^C, suggesting an independent evolution of organelle methylation in animals and plants [52]. The most frequently occurring methylcytidines are found at positions 48 and 49 at the junction of the VL and TSL. Unlike the m^1^A_9_, m^1^G_9_, m^2^G_10_ and m^2^_2_G_26_ methylations at positions 9, 10, and 26, respectively, on the 5’-side of tRNAs core at the junction of the accepting stem and D-stem, and the DSL and ASL stems, the methyl group of m^5^C_48_ and m^5^C_49_ located at the 3’-side of the tRNA’s core are not on the Watson–Crick H-bonding face of the nucleobase, and therefore do not physically interfere with canonical base pairing. RNA modification to m^5^C is important in regulating RNA metabolism and promotes tRNA stability and protein synthesis in eukaryotes and prokaryotes [52,53]. The m^5^C modification is catalyzed by RNA-specific m^5^C-methyltransferases using the methyl donor, S-adenosyl-l-methionine (Ado-Met). In yeast *Saccharomyces cerevisiae*, a newly identified multisite-specific yeast tRNA methyltransferase (Trm4) was found to catalyze m^5^C modification at four different positions in tRNA [54]. NSUN2 (cytosine-5 RNA methyltransferase) catalyzes the methylation of cytosines at C_34_, C_48_, C_49_ and C_50_ [53,55,56]. Although m^5^C is present in at least 34 different yeast tRNA species, in tRNA^Leu^_CAA_ it is uniquely found at the wobble position as well as at position 48 in the VL. Upon cellular oxidative stress, however, the distribution of m^5^C in the tRNA is dynamically changed, with a significant increase at the wobble position and a concomitant decrease at position 48, indicating a specific response to the stress [57].

In protozoan parasites, most tRNAs display at least one m^5^C at the junction between the VL and the TSL. The m^5^C modification leads to stabilization of the tRNA core in eukaryotes and archaeal tRNAs, but not in eubacteria [58]. In *Trypanosoma brucei,* methylation of tRNA^Asp^_GUC_, tRNA^Gly^_GCC_, tRNA^Val^_AAC_ and tRNA^Tyr^_GUA_ was observed in residues at the TSL/VL junction including C_48_ and C_49_. In tRNA^Tyr^_GUA_ both un-spliced and spliced molecules contained m^5^C_48_, indicating cytosine methylation can precede tRNA splicing [59], as had been reported for core modifications of bacterial precursor tRNAs.

In all tRNAs, nucleoside 48 in the VL forms a non-canonical base pair with the nucleoside 15 in the D-loop known as the ‘Levitt pair’ (Figure 3a,b) [60]. Instead of a canonical Watson–Crick base pairing, nucleosides 15 and 48 assume a reverse Watson–Crick (RWC) geometry or a *trans* arrangement. The RWC *trans* configuration brings together the DSL and VL, joining the two helical domains of tRNA [61] (Figure 3a). In some tRNAs, one of the nucleosides in the Levitt pair, G_15_○C_48_, is modified. (The open circle, ○, denotes a non-canonical base pair.) The G_15_○m^5^C_48_ Levitt pair occurs in 26% of tRNA sequences found in databases [62]. However, the physicochemical contribution of the methyl group has not been understood. The methyl of m^5^C_48_ increases the hydrophobic character of the nucleoside pair and may contribute to base stacking. In archaeal tRNAs, G_15_ is found to be modified to 7-formamidino-7-deazaguanosine (fa7d7G or ADG), known as archaeosine, G^+^ (Figure 2c) [27,63]. The imidino side chain of archaeosine has a distributed positive charge among the resonance of its conjugated double bonds [62,63]. Positive charges at the junction of the amino acid accepting stem and the DSL are important to the transcription and subsequent proper tertiary folding of tRNAs [42]. Both metal (Mg^2+^ ion) binding and chemical modifications have been suggested to be factors stabilizing the RWC geometry of the Levitt base pair [62]. The Levitt pair interaction forms the innermost base-pair in the augmented D-stem [64] and is usually between a purine nucleoside at position 15 and a pyrimidine at position 48 which form a non-Watson–Crick hydrogen bonding due to the *trans* orientation of the glycosidic bonds [27,65]. However, a small number of species including *E. coli* tRNA^Cys^ contain the unusual G_15_○G_48_ pairing (Figure 3c). Crystal studies have shown the G_15_○G_48_ pairing to have the same *trans* orientation as the conventional Pu_15_○Py_48_ Levitt pair [66].

The *E. coli* tRNA^Cys^ core containing the unusual G_15_○G_48_ base pair joins the D- and V-loops and is very sensitive to alterations. This unusual Levitt pair may be important for aminoacylation by cysteinyl-tRNA synthetase, CysRS [65,67]. Substitution to the more conventional G_15_○C_48_ pairing in tRNA^Cys^ destabilized the tRNA’s core and decreased the catalytic efficiency of aminoacylation (*k_cat_/K_m_*) by nearly 100-fold [67]. In contrast, the same substitution in *E. coli* tRNA^Ala^ had minimal effect on aminoacylation [68]. The sensitivity of tRNA^Cys^ to the Levitt pair substitution may be due to the unusual H-bonding between the O6 of G_15_ and N1 and N2 of G_48_ (Figure 3 b and c) [66]. The interaction of G_15_ O6 with the G_48_ N1 and *N^2^* is different from the G_15_○C_48_ base pairing of other tRNAs. In other tRNAs the N1 and N2 of G_15_ are paired with O2 and N3 of G_48_, respectively [25]. The triple base stacking of A_46_○(U_8_●A_14_) below the Levitt pair appears to be unique to this tRNA and along with several layers of base pairs above and below G_15_○G_48_, provides additional stacking interactions to stabilize G_15_○G_48_ in tRNA^Cys^. In addition, tRNA^Cys^ contains the A_9_○(A_13_●A_22_) base triple. The 9○(13●22) pairing is rare and only found in *Thermus thermophilus* tRNA^Ser^ as a G_9_○(G_13_●A_22_) base triple [69]. Variations in the VL, alone and not the loops of the DSL or TSL rescued the kinetics of aminoacylation in the G_15_○C_48_ mutant of tRNA^Cys^ [25].

### 2.2. Methylations of the Ribose 2’-OH and Nucleobase

Methylated nucleosides contribute to the stability of tRNA. Methylation can either occur at the 2’-hydroxyl of the ribose or on the nucleobase itself. The 2’-*O*-methylation (Figure 2e) induces the C3’-*endo* ribose conformation for both purine and pyrimidine nucleosides, stabilizing A-type helical regions within tRNA [45,70,71]. In observations of 2’-*O*-methyluridine 3’-monophosphate (Ump), the C3’-*endo* conformation was found to be 0.67 kcal/mol more stable than the C2’-*endo* conformation; conversely, the C2’-*endo* conformation was slightly favored in the uridine 3’-monophosphate (Up) (Figure 2) [71]. Again, steric interactions are responsible for preference of Ump for the C3’-*endo* conformation. Repulsion between the 3’-phosphate and the 2’-*O*-methyl group forces the 2’-*O*-methyl group to orient itself toward the uracil base, in turn increasing steric repulsion between the 2’-*O*-methyl group and the 2-carbonyl group [71]. Similar to the effect of the 2-thio modification on tRNA thermal stability, the 2’-*O*-methyl modification is favorable to thermophilic organisms whose tRNA structure must withstand high temperatures. Alternatively, the 2’-*O*-methyl modification has been proposed to protect tRNA from degradation at high temperatures; the tRNA would otherwise be increasingly susceptible to ribonuclease attack [72].

The incorporation of the 2’-*O*-methyl modification into thermophilic tRNA in vivo is temperature dependent. *Bacillus stearothermophilus* cultured at 70 °C experienced a three-fold increase in 2’-*O*-methylribose moieties in tRNA compared to the cultures grown at 50 °C [72]. Similar results were obtained for *Thermus thermophilus* HB27 cultured at 80 °C and 50 °C in which the 2’-*O*-methylguanosine (Gm) content of tRNA in particular was compared [73]. However, *T. thermophilus* tRNA (guanosine-2’) methyltransferase activity rather than tRNA content of Gm was affected by the temperature difference. Thus, the thermophilic enzyme may have evolved to be activated by high temperatures [73]. Otherwise, at lower temperatures, *T. thermophilus* tRNA is sufficiently structured without an abundance of the 2’-*O*-methyl modification and methyltransferase activity is redundant.

The presence of methylated nucleobases, including *N*^1^-methyladenosine (m^1^A) and *N*^7^-methylguanosine (m^7^G) within tRNA proves to be beneficial to *T. thermophilus* as well. *T. thermophilus* HB27 lacking tRNA (m^1^A_58_) methyltransferase activity displayed a thermosensitive phenotype at 80 °C [74]. *T. thermophilus* HB8 strain subjected to disruption of the tRNA (m^7^G_46_) methyltransferase (TrmB) gene also experienced a growth defect at temperatures above 70 °C [75]. Moreover, tRNA from the Δ*trmB* mutant strain was determined to increase the rate at which Gm_18_, m^1^G_37_, and m^1^A_58_ are formed by their respective modification enzymes [75]. By promoting the formation of other stabilizing modifications, m^7^G_46_ is implicated in an intricate network of tRNA modifications [75].

The aforementioned methylated nucleobases are not exclusive to prokaryotic, thermophilic tRNA. The methyltransferase encoded by the *WDR4* gene, for example, is responsible for the m^7^G_46_ modification within human tRNA. Mutation of arginine-170 to leucine on the WDR4 protein results in decreased levels of m^7^G incorporated into tRNA, and is correlated with microcephalic primordial dwarfism [76]. The mechanism by which the diseased state of microcephalic dwarfism appears remains under investigation. The m^1^A_58_ modification is also common to tRNA from all domains of life [51]. Within *S. cerevisiae* Δ*gcd10* mutants, the m^1^A tRNA modification was undetectable and the amount of mature initiator methionyl-tRNA (tRNA_i_^Met^) was reduced [77]. However, neither syntheses of pre-tRNA_i_^Met^ nor elongator methionine tRNA (tRNA_e_^Met^), which also contains the m^1^A_58_ modification, were affected. Thus, the m^1^A_58_ modification has been proposed to play a role in the maturation of pre-initiator tRNA_i_^Met^, preserving its unique tertiary structure in comparison to the structure of tRNA_e_^Met^ [77]. The introduction of a positive charge on A_58_, such that the A_58_○U_54_ reverse Hoogsteen base pair remains undisturbed, is thought to be the stabilizing force [16,78]. In fact, both the positively charged m^7^G_46_ and m^1^A_58_ stabilize their respective base triplet and pairing interactions. Positively charged m^7^G_46_ and m^1^A_58_ are conserved across a substantial number of cytosolic tRNAs. In the gas phase, C_13_●G_22_○m^7^G_46_ is stabilized by 17.4 kcal/mol while T_54_○m^1^A_58_ and U_54_○m^1^A_58_ are stabilized by 6.8 kcal/mol and 6.2 kcal/mol, respectively, as compared to their unmodified analogs [79].

### 2.3. Uridine Modifications Affecting Core Structure

Ribothymidine, T or m^5^U, is commonly found in the T-arm of various tRNAs. The added methyl group increases the hydrophobicity of the base, reducing its ability to be a hydrogen donor in hydrogen bonding with adenosine. In comparison, uridine can weakly base pair with any of the conventional nucleosides [80]. Nucleobase methylations not located on the Watson–Crick face, such as m^5^C, do not interfere with canonical base pairings. However, they also have been reported to enhance the stacking capability of nucleosides, thus enhancing the overall stability of especially the tertiary RNA structure. The temperature at which the initiator bacterial tRNA^fMet^ structure with T_54_ is half denatured, the melting temperature or T_m_, was found to be 6 °C higher than the same tRNA with U_54_ and 13 °C higher with 2-thioribothymidine, s^2^T_54_, than with the unmodified U_54_ [81].

#### 2.3.1. Dihydrouridine

The reduction of the double bond linking C5 and C6 of uridine yields the dihydrouridine modification (Figure 2b). The dihydrouridine loop (D-loop) is a conserved region found in tRNAs in virtually all living systems. The presence of D within the D-loop plays an instrumental role in giving tRNA both its secondary and tertiary structure [82,83,84,85]. The crystal structure of the nucleoside D was elucidated during the early 1970s. Within the crystalline network, D does not participate in base stacking due to its loss of aromaticity. The ribose of D was determined to favor the C2’-*endo* conformation [86], whereas that of the parent U favors neither the 2’- or 3’-*endo* conformation. The change in energy between the C3’-*endo* and C2’-*endo* conformations of pyrimidines is nominally only 0.5 kcal/mol. The small difference in energy between the C3’-*endo* and C2’-*endo* conformations allows for flexibility of the ribose but does not appear to be the case for modified nucleosides. In fact, the saturation of the C5=C6 double bond of the nucleobase gives rise to the C2’-*endo* conformation of the ribose; whereas methylation of the 2’-OH stabilizes the C3’-*endo* pucker. The interaction between a lone pair of O4’ and the pi antibonding orbital of the C5=C6 double bond of unmodified uridine is absent in D [87,88]. The resulting increase in dihedral angle χ subsequently allows the ribose to assume the C2’-*endo* conformation [87]. However, in solution, D was observed to induce a hairpin loop in the DSL reportedly due to its ability to engage in base stacking on only one side [83]. Furthermore, a DSL lacking D at position 16 was unable to achieve a stable hairpin, instead interconverting between various conformations [83].

In a 2C_9_N_2_O_6_H_14_⦁H_2_O unit cell of a D crystal, one D molecule adopts a *gauche*–*trans* conformation around its C4’–C5’ bond, while the other preferentially adopts a *trans*–*gauche* conformation over a gauche–gauche conformation [89]. Conversely, unmodified uridine strongly favors the *gauche*–*gauche* conformation over the *trans*–*gauche* and *gauche*–*trans* conformations [90]. Although the D residue is asymmetric, both the *gauche*–*trans* and the *trans*–*gauche* conformations allow for the formation of loop structures within tRNA, while the *gauche–gauche* conformation of uridine is typically found within double helical structures [89]. D has the potential to destabilize double-stranded, helical RNA due to its ribose conformation. When substituted into 21-nucleoside RNA duplexes, D disrupted the A-type helical structure and decreased the melting temperature of the duplexes [91].

In yeast tRNA^Phe^, among its 76 nucleosides there are twelve that assume the C2’-*endo* conformation. The 12 nucleosides with C2’-*endo* conformations occupy single-stranded loops and stretches. The single stranded regions of tRNA, though not involved in secondary structure motifs, are involved in tertiary interactions [92]. The majority of nucleosides within both yeast tRNA^Phe^ and tRNA^Asp^ that adopt the C2’-*endo* conformation (seven out of twelve and seven out of ten, respectively) are located within or just outside of the D-loop. The minority of nucleosides adopting the C2’-*endo* conformation and located other than in the D-loop in tRNA^Asp^ are afforded a greater degree of flexibility, resulting in a region in which base intercalation occurs [92,93]. Evidently, the inclusion of D within the primary sequence of tRNA directly affects the arrangement of characteristic secondary and tertiary interactions.

The prevalence of D within tRNA is especially advantageous for psychrophiles, organisms that optimally grow at temperatures lower than 15 °C and cannot grow at temperatures above 20 °C [94]. At these low temperatures, psychrophiles require modifications, such as dihydrouridine, that enhance tRNA flexibility. The tRNA digests of three psychrophilic bacteria were analyzed by liquid chromatography-mass spectrometry (LC-MS): ANT-300 (an unnamed species of *Moritella*) and *Vibrio* sp. strains 5710 and 29-6 [95]. The tRNA digests of the three psychrophilic bacteria contained between 1.33 and 1.46 times the D content of mesophilic *E.* *coli* tRNA digests [82,95]. Interestingly, the T_m_ of the psychrophilic tRNA (~77 °C) did not differ significantly from the T_m_ of *E. coli* tRNA (75 °C), suggesting that D contributes to local rather than global flexibility [95,96]. Compared with psychrophiles, psychrotolerant organisms undergo optimal growth at temperatures above 15 °C; however, these organisms are viable at lower temperatures as well [94]. The first study of tRNA modifications in psychrotolerant Archaea centered on *Methanococcoides burtonii* [45]. Notably, the presence of D was confirmed within *M. burtonii* tRNA, while D was not detected within the archaeal hyperthermophile *Stetteria hydrogenophila* [45].

Conversely, thermophiles must contain modifications that confer stability onto their tRNA, effectively the opposite to the destabilizing function of D. Thermophilic tRNA containing 2-thioribothymidine at position 54 (s^2^T_54_) is resistant to denaturation (Figure 2b). The C=S bond of s^2^T is longer than the C=O bond of unmodified T, and the van der Waals radius of sulfur is larger than that of oxygen [97]. The increased steric repulsion between the thiocarbonyl of the nucleobase and the 2’-hydroxyl of the ribose promotes the C3’-*endo* conformation [97]. Additionally, the conformation around the C4’–C5’ bond is almost exclusively *gauche–gauche* [97]. As previously stated, the *gauche–gauche* conformation is characteristic of helical regions of RNA [89]. Two species of tRNA^Ile1^ have been identified in *Thermus thermophilus* HB8 grown at 65 °C [98]. The species, tRNA^Ile1a^ and tRNA^Ile1b^, have identical sequences with the exception of the nucleoside at position 54: tRNA^Ile1a^ contains the modified nucleoside s^2^T_54_ while tRNA^Ile1b^ contains the unmodified T_54_ [98]. The T_m_ of tRNA^Ile1a^ (86.2 °C) was 2.9 °C higher than that of tRNA^Ile1b^ (83.3°C) [98]. Here is an instance in which a single modification has a profound impact on the thermostability of the entire tRNA molecule.

#### 2.3.2. Pseudouridine

Pseudouridine (Figure 2b) is the most frequently occurring modified nucleoside in RNA [1,99,100]. It is found in all three domains of life as well as in the organelles [101]. Furthermore, Ψ is found in various RNAs including tRNA, rRNA, mRNA, and some small RNAs such as snoRNA [99,101,102]. Existing uridines are modified into Ψ by pseudouridine synthases via a glycal intermediate [103,104]. Ψ is an isomer of uridine, where instead of N1 of the base being linked to the sugar, the C5 of the base is connected to the sugar. Pseudouridine synthetase refashions U by cleaving the N^1^–C1’ glycosidic bond and produces a C5–C1’ bond without releasing the uracil nucleobase in forming Ψ residues in tRNA transcripts. The modified glycosidic linkage resulting in C5–C1’ bond slightly alters the chemical properties of the nucleoside that can cause drastic differences in structure and function. In tRNA, Ψ is found at various positions and three-dimensional environments, depending on both the isoacceptor as well as the organism (or organelle) itself [105]. Nuckear magnetic resonance (NMR) studies of Ψ compared to U have shown that in the isomer the imino N^1^-H proton is in slow exchange with water, a property commonly associated with specific structural environments. The slow exchange of the imino N^1^ with water is an intrinsic property of pseudouridine rather than an effect caused by base pairing since the slow exchange of the imino proton has been shown to occur in a variety of contexts [106]. While Ψ has been found at many positions of the anticodon stem and loop domain in a functional role, its occurrence in tRNA is most commonly found in a more structural role, especially in the TSL and certain positions in the ASL. Ψ is historically and most commonly associated with position 55 of the TSL in the sequence TΨCG.

Perhaps the most striking effect of Ψ appears to be its ability to enhance RNA stabilization through sugar puckering, where the RNA helix is stabilized by favoring the C3’-*endo* pucker [105,106]. The altered puckering (Figure 2f), has been linked with enhanced stacking ability [105,106], explaining the increased stability pseudouridine modifications provide [91,106]. In recent computational studies, Ψ did not strongly favor either C2’-*endo* or C3’-*endo* puckering, but a clear energy minimum was observed for the *anti*-conformation of the C5–C1’ between the base and ribose [107]. The same effect has been observed in NMR studies of Ψ alone, whereas in an oligonucleoside the C3’-*endo* puckering is apparent [106]. The energy barriers between C2’-*endo* or C3’-*endo* puckering and between the *syn* and *anti*-glycosidic bond angles for uridine and Ψ must be low for all combinations have been observed and may depend on the experimental conditions, sequence context and the biophysical approach taken [108,109]. In particular, the context in which these nucleosides appear in an RNA sequence and its secondary and tertiary structures are most likely to influence the ribose backbone and the glycosidic bond conformations taken at any particular site. Computational as well as NMR studies indicate that Ψ induces C3’-*endo* puckering in its neighbors, therefore enhancing the rigidity of the local structure, and hence stabilizing the A-form RNA [106,107]. The inherent ability of Ψ to stabilize RNA structures through enhanced C3’-*endo* sugar puckering and enhanced base stacking compared to uridine has been extensively demonstrated experimentally through circular dichroism (CD), NMR, and UV-melting studies [106,110].

Studies on pseudouridine synthetase (Pus1) from *S. cerevisiae* indicate the importance of tertiary base pairing in Pus1 tRNA recognition. The presence of a DSL and the non-canonical G_26_○A_44_ mismatch pair near the target uridine 27 is important for Pus1 tRNA high affinity recognition, resulting in an increase in association rate constant (K_a_) by a factor of 100 and commensurate decrease in the equilibrium constant for the reaction [111]. In addition to enhancing the local stability of its neighboring nucleosides, Ψ contributes to the stability of tRNA’s three-dimensional structure. The D-loop and T-loop interact in the tertiary structure through several base pairs, including G_15_●C_48_ (Figure 3) and G_19_●C_56_ and G_18_○Ψ_55_ (not shown in Figure 3). The m^1^A_58_○T_54_ base pair is sandwiched between the two aforementioned base pairs where the modified nucleosides are crucial for enhanced structural stability. The stability is further increased if T_54_ is replaced by s^2^T_54_ [81].

## 3. Modifications Impacting Decoding

### 3.1. Modifications at Position 34

The most numerous, and most chemically varied, of the post-transcriptional modifications to tRNA occur in the ASL domain, particularly at positions 34, the first position of the anticodon and position 37, 3’-adjacent to the anticodon [5,112]. Modifications of positions 34 and 37 have been implicated in a wide range of functional roles, including alteration of tRNA conformation and thermal stability [113,114,115,116,117,118], enhancement of ribosome binding [113,115,119,120,121], promotion of mRNA translocation during translation [121], augmentations of mRNA decoding specificity [117,122,123,124,125], maintenance of the mRNA reading frame [126,127,128,129] and the pre-structuring of the ASL into the canonical U-turn structural motif for ribosomal A-site entry [20,117,126,130].

Position 34 in the tRNA ASL is sometimes referred to as the “wobble” position in reference to Francis Crick’s 1966 Wobble Hypothesis [131]. The Hypothesis states that a guanosine, uridine or inosine occupying position 34 has the ability to base pair to two or three different nucleosides. This ability of nucleosides at position 34 of the anticodon to bind to non-canonical bases or “wobbling” addresses the degeneracy of the genetic code. The concomitant existence of fewer tRNAs than possible mRNA codons, means a single tRNA species decodes more than one synonymous codon differing in the identity of the residue at the third position. Twenty-five years later, the Modified Wobble Hypothesis expanded Crick’s original postulate. The Modified Wobble Hypothesis took into account evidence of codon recognition being restricted or expanded by the presence of a chemical modification to the wobble nucleoside, allowing the anticodon–codon interaction to be fine-tuned [9].

The presence or absence of modifications to position 34 uridines displays a particularly clear effect on wobble decoding. In certain cases, an unmodified position 34 uridine permits even broader codon recognition than the Wobble Hypothesis predicts; many mitochondrial and plastid tRNAs with a wobble uridine can decode all four codons in a single codon box [132,133,134,135]. In general, however, cytosolic tRNAs across species require modification to a wobble position uridine in order to recognize any nucleoside in the third position of the mRNA codon (other than its exact cognate A). Eighty-four percent of the sequenced cytosolic tRNAs carry modifications at position 34. Modifications at position 34 have been shown to restrict or expand the decoding ability of the tRNA [115,136,137,138,139,140,141,142,143].

Unexpectedly, most post-transcriptional modifications to wobble nucleosides occur at position 5 of a pyrimidine, on the opposite site of the nucleobase from the Watson–Crick face [5,131]. Modification to the Watson–Crick face is predicted by the original Wobble Hypothesis to facilitate wobble pairings. Modifications other than those to the Watson–Crick face must, therefore, affect the chemistry at the anticodon–codon interface indirectly, by influencing the overall conformation and structure of the nucleoside. One structural effect of 5-position modifications is the biasing of the ribose sugar moiety of the nucleoside toward either a C3’- or C2’-*endo* conformation; the C3’- or C2’-*endo* sugar pucker have been shown to restrict or expand, respectively, the overall decoding capacity of the tRNA [7,9,144,145,146]. 

The restriction of decoding is frequently coupled to substitution of the 2-position keto group with a thio group along the Watson–Crick face. Modification of the Watson–Crick face with a thio group may affect the hydrogen bonding chemistry of uridine as a proton donor in addition to stabilizing the C3′-*endo* conformer [144,145,147,148]. For example, modifications in the 2-thio-5-methyluridine (xm^5^s^2^U) family at position 34 bias the nucleoside toward its C3′-*endo* form due to the large van der Waals radius of the sulfur atom. The larger van der Waals radius of the sulfur atom sterically repels the ribose 2′-oxygen atom. The 2-thiolated modified uridines typically recognize only adenosine as the third letter of the codon [146,149]. By contrast, modified nucleosides in the 5-hydroxyuridine (xo^5^U) family can adopt the C2′-*endo* as well as the C3′-*endo* form, and they expand wobble recognition to include adenosine, guanosine, and uridine [146].

Structural studies also indicate that the larger modifications to position 34 uridines also operate to directly influence the stability of base pairs at the anticodon–codon interface. The 5-taurinomethyluridine (τ^5^mU_34_) modification is a member of the xm^5^U family of modifications. The τ^5^mU_34_ modification is found in several mitochondrial tRNAs (Figure 2b) and its absence from tRNA^Leu(UUR)^ and tRNA^Lys^ is implicated in MELAS (mitochondrial encephalopathy, lactic acidosis and stroke-like episodes) and MERRF (myoclonic epilepsy with ragged red fibers) syndromes [150,151,152]. Together with its orthologous *E. coli* modification 5-carboxymethylaminomethyluridine (cmnm^5^U_34_), the τ^5^mU_34_ modification has been shown to play a role in codon decoding by stabilizing U_34_●G_3_ wobble base pairs [120]. Both crystallographic and computational studies of the τ^5^mU_34_-modified ASL of mitochondrial tRNA^Leu^_UAA_ have revealed stabilized hydrogen bonding interactions between the sulfonic acid and secondary amine moieties of the modification and nucleosides U_33_, A_35_ and A_36_, as well as adoption of a non-Watson–Crick U_34_●G_3_ geometry thought to enhance stacking [120,153]. Likewise, 2-thiouridine geranylation (Figure 2b) in bacteria facilitates tRNA^Glu^_UUC_ and tRNA^Lys^_UUU_ formation of stable Watson–Crick U_34_●G_3_ wobble base pairs at the anticodon–codon interface [154,155]. The large hydrophobic terpene geranyl group covalently modifies the position 2 thio group of 2-thiouridine inducing a loss of protonation at the N3 atom of U_34_ [156].

Adenosine in position 34 of the anticodon is nearly universally modified to inosine an analog of guanosine. Inosine binds cytosine in the third position of the codon, as well as uridine and adenosine according to the predictions of the Wobble Hypothesis [131]. The prevalence of this A_34_-to-I_34_ modification is so complete that only four exceptions are noted: tRNA^Thr^_AGU_ of *Mycoplasma capricolum* and *Mycoplasma mycoides*; and mt tRNA^Arg^_ACG_ of *S. cerevisiae* and *A. suum* [157,158,159,160,161,162]. In eukaryotes, all four-fold degenerate codon boxes are decoded by at least one tRNA isoacceptor that contains a wobble-position inosine.

The increased spatial extent of an inosine–adenosine (I_34_○A3) pair in the wobble position of the codon on the ribosome led to significant early speculation on the preferred geometry adopted by this purine○purine pair. Crick originally proposed traditional Watson–Crick base pairing geometry for the I_34_○A3 pair without addressing the additional 1.6 A of space required of an inosine–adenosine base pair [131]. An alternative hypothesis suggested that the inosine adopts a *syn* *N-*glycosidic bond conformation, exposing its Hoogsteen face for binding to A3 rather than its canonical Watson–Crick chemistry and better allowing the base pair to occupy a space intended for a smaller purine●pyrimidine interaction[149]. However, an x-ray crystal structure of inosine-modified *E. coli* tRNA^Arg^_ICG_ bound to its wobble CGA codon in the A-site of the 30S ribosome unambiguously demonstrated that I_34_○A3 adopts traditional Watson–Crick geometry. The broader spatial extent of an I_34_○A3 pair is accommodated by a compression of the I_34_ ribose and phosphate moieties between C1′ and P of that residue, where the β (P-O5′-C5′-C4′) torsion angle, in particular, changes from about 180° to −37.8° [163].

Apart from its role in tRNA decoding, A-to-I deamination also plays an important role in mRNA editing, with hundreds of millions of examples in eukaryotic transcriptomes [164]. In the context of tRNA decoding of mRNA codons, A-to-I editing of mRNA coding regions alters the coding potential by tRNAs. Where inosine resides in the codon, wobble base pairing occurs in a non-traditional reverse direction; accuracy and efficiency of decoding could potentially be affected by tRNA modifications of the anticodon domain. The A-to-I deamination reaction in mRNA is catalyzed by a specialized family of adenosine-deaminases-acting-on-RNA proteins (ADARs), whose A-to-I editing function acts on double-stranded coding regions of mRNA as well as non-coding introns, the 3′-untranslated regions of mRNAs and in microRNAs [165]. The A-to-I editing can alter the splicing profile and miRNA-mediated gene silencing of altered transcripts, providing another level of control of gene expression [164,165]. Mutations in ADARs have been associated with human pathology, including cancer, neurological disorders and defects in innate immunity [166].

**Figure 4:**
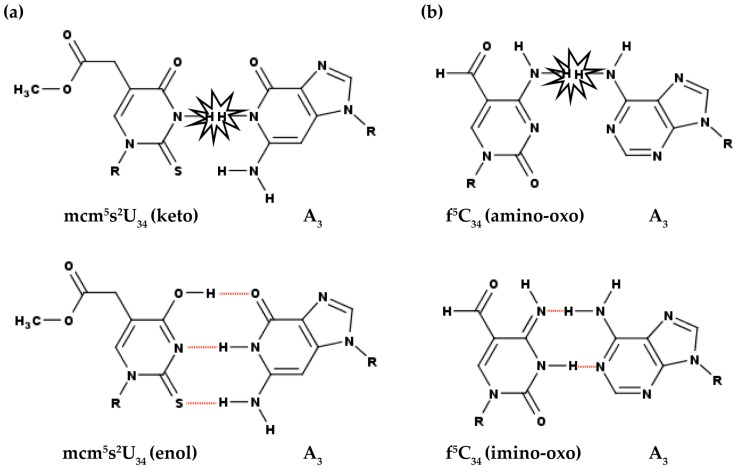
Modification-induced tautomerism allows the wobble residue to base pair with a non-cognate residue in the third codon position. (**a**) The keto form (Upper) of mcm^5^s^2^U_34_ encounters steric hindrance when forming a Watson–Crick base pair with adenosine; while the enol form (Lower) allows for favorable hydrogen bonding; (**b**) The common amino-oxo form (Upper) of f^5^C_34_ is sterically prohibited from forming a Watson–Crick base pair with adenosine; while the imino-oxo form (Lower) hydrogen bonds favorably.

Modifications at position 34 have also been shown to enable biologically relevant mismatch base pairing, particularly at the wobble position, by permitting tautomerism of the modified nucleobase (Figure 4). In human and *E. coli* tRNA^Lys^_UUU_, a 5-methoxycarbonylmethyl-2-thiouridine or 5-methylaminomethyl-2-thiouridine modification at position 34 (mcm^5^s^2^U_34_ or mnm^5^s^2^U_34_), respectively, allows the tRNA to recognize the non-cognate wobble AAG codon. The modification has been shown in crystal structures to shift the uridine keto–enol equilibrium to the enol form due to an alteration of the electronic structure of the uridine ring. The enol tautomer permits the G●U base pair to maintain Watson–Crick geometry in the ribosomal A-site and avoid steric clashes (Figure 4a; [167,168]). A similar keto–enol tautomerization is observed for *E. coli* tRNA^Val3^_UAC_ with cmo^5^U_34_. The modification also permits the wobble uridine to adopt its enol form for Watson–Crick mismatch base pairing with a guanosine [136]. Tautomerism enables a G●U mismatch in the wobble position of the codon–anticodon duplex to adopt the geometry of a Watson–Crick pair. The modification-facilitated geometry of a Watson–Crick base pair is thought to allow such a G●U mismatch to escape discrimination by the translational fidelity mechanism [168,169]. In crystallographic studies of human mitochondrial tRNA^Met^_CAU_ bound to the non-cognate AUA codon on the ribosome, a 5-formylcytidine modification at position 34 (f^5^C_34_) also appears as the rare imino-oxo, (rather than the common amino-oxo) tautomer of cytidine. An NMR study of rare, transient mismatch base pairs involving unmodified A, G, C and U has shown that anionic, as well as tautomeric forms, can occur and be important to the chemistry and structure of RNA [170]. Observation of a possible imino-oxo tautomerization suggests again a role in vivo for the modification to shift the tautomeric equilibrium toward the imino form. In the imino form, the wobble f^5^C_34_○A3 mismatch base pair is stabilized in Watson–Crick geometry (Figure 4b) [171]. Thus, in order for tRNA to effectively fulfill its role in an expended wobble recognition, certain position 34 modifications facilitate in vivo the Watson–Crick geometry of anticodon–codon mismatches through tautomerization and possibly anionic forms.

### 3.2. Modifications at Position 37

Modifications at position-37 contribute both stereochemistry and favorable energy to formation of the Watson–Crick base pair. Accuracy and efficiency in vivo is dependent on a number of factors: codon bias and usage; energetics of base pairing (e.g., A●U, U●A vs. G●C and C●G); codon context or nearest neighbor nucleosides and codons; tRNA species concentration and thus competition for codon. Of considerable importance to accuracy and efficiency are the post-transcriptionally modified nucleosides at position-37, 3′-adjacent to the anticodon, as well as at the anticodon wobble position-34 [126,130,170,172,173,174]. There are a number of significant advantages to modification of the invariant purine nucleoside at position-37. Modification of purine-37 negate cross-loop base pairing. Early evidence points to the closing of the unmodified anticodon loop by H-bonding [175]. An open anticodon loop facilitates and frees the last two anticodon bases for canonical base pairing, and the first base for canonical and wobble pairings. Hydrophobic modifications at position-37 are supportive of the 3′-stack of the anticodon facilitating anticodon presentation to the codon [167,175,176,177]. The modifications of A_37_ produce a hydrophobic platform. The hydrophobic platform is particularly noteworthy for it envelops the first anticodon–codon base pair of those tRNAs responding to codons starting with A1 [130,178,179]. Position-37 purine modifications help pre-structure, order, the anticodon domain for the recycling of the tRNA without investment of energy and chemistry to regain the conformation and dynamics for the integrity of codon recognition [114,174]. The physicochemical contributions of modified purine nucleosides-37 collectively maintain the translational reading frame in all kingdoms of life by supporting correct formation of the critical first base pair, A_36_ or U_36_ hydrogen bonding to U1 or A1 respectively, of the mRNA codon [127,180,181,182]. There are human disease associations to mutations in the enzymes responsible for the position 37 modifications. For instance, mutations of the human protein CDK5 regulatory subunit associated protein 1-like 1 (Cdkal1), the enzyme responsible for the ms^2^ modification of *N*^6^-threonylcarbamoyladenosine, t^6^A_37_ (Figure 2a), are associated with lack of processing of proinsulin and decreased secretion of the mature insulin [183]. Interestingly, a new reactive species, cysteine hydropersulfide (CysSSH) has been reported as substrate and thio-donor for this enzyme with suppression of CysSSH production associated with a decrease in insulin secretion [184]. At least one of the purine-37 modification enzymes, human tRNA isopentenyltransferase, TRIT1, has a direct consequence on human health for it is a tumor suppressor [181], whereas modification of G_37_, 3′-adjacent to A_36_ in phenylalanine tRNAs, may actually promote frameshifting in some codon contexts [185].

When the first codon base is U1 or A1, the responding tRNA anticodon with an A_36_ or U_36_, respectively, has a 3′-adjacent modified A_37_. The modification can be of extraordinary chemistry and structure, such as *N*^6^-isopentenyladenosine, i^6^A and its derivatives (e.g., 2-methylthio-*N*^6^-isopentenyladenosine, ms^2^i^6^A); *N*^6^-threonylcarbamoyladenosine, t^6^A and its derivatives (e.g., 2-methylthio-*N*^6^-threonylcarbamoyladenosine, ms^2^t^6^A). Phenylalanine tRNAs, which respond to codons beginning with U, have G as the 3′-adjacent purine-37 instead of an A_37_. Still, tRNA^Phe^ G_37_ is extensively modified. The G_37_ of tRNA^Phe^ from Archaea, Eukarya and even mitochondria is modified to a tricyclic wyosine/wybutosine, imG/yW, (3*H*-Imidazo[1,2-α]purine-7-butanoic acid, 4,9-dihydro-α-[(methoxycarbonyl)amino]-4,6-dimethyl-9-oxo-3-β-d-ribofuranosyl-, methyl ester, (α*S*)-) or a derivative [186,187], the biosynthesis of which begins with a simple N1 methylation [188].

The elaborate *N^6^* modification of A_37_ with i^6^ or t^6^, and of G_37_ as imG_37_ or yW_37_ deny stable cross-loop hydrogen bonding by position 37 nucleosides. All tRNAs responding to codons beginning with U1 or A1 have the invariant U_33_ at the U-turn otherwise capable of cross-loop H-bonding between U_33_ and A/G_37_ [118,175]. The rotational freedom of t^6^A about the N11–C12 bond results in steric hindrance, preventing the incorporation of the nucleoside into the helix and therefore, abrogating hydrogen bonding with U_33_ [179]. The open loop afforded by the modification is necessitated by the geometry of Watson–Crick base pairing of the anticodon to the codon. Both t^6^A_37_ and imG/yW_37_ have either non-covalently or covalently formed tricyclic bases. The tricyclic imG/yW_37_ are seen as platforms above the U_36_-A1 or A_36_-U1 anticodon–codon base pairs [167]. The cyclized ester of t^6^A_37_ (ct^6^A_37_) in the oxazolone ring form has been found in a number of species and is a stable derivative enzymatically closed [189,190]. However, a hydantoin isoform was determined by x-ray crystallography [191]. The hydantoin form may be of considerable consequence to the decoding of mRNA codons beginning with A. It is found in *E. coli* tRNA and may not be able to lend support to the first anticodon–codon base pair due to possible twisted conformations that are not co-planar with the adenine nucleobase. Position 37 modifications contribute considerable energetic stability to the first base pair through van der Waals forces and hydrophobic interactions [167,192,193]. In contrast to t^6^A_37_, yW_37_ in some eukaryotes may, in the context of particular mRNAs, contribute to frameshifting, whereas the unmodified, hypomodified, nucleoside will maintain the translational reading frame [185].

tRNAs with anticodons ending in G_36_ or C_36_ respond to codons starting with C1 or G1 and may or not have a modified a A_37_ or G_37_. The G_36_●C1 and C_36_●G1 first base pairs with additional hydrogen bonding are energetically more favorable in comparison to U_36_●A1 and A_36_●U1. In addition, the purine-37 stacks well with G_36_●C1 and C_36_●G1 supporting the Watson–Crick base pairing. However, methylation of the N1 of G_37_ suppresses +1 shifts in the translation reading frame [194,195]. tRNA species for leucine, proline and arginine that respond to codons beginning with C1 are modified to m^1^G_37_. tRNA^Pro^_UGG_ with the anticodon UGG is particularly susceptible to +1 frame shifts, and the tRNA^Pro^_GGG_ species requires m^1^G_37_ and the aid of the translational factor EF-P (elongation factor P) to suppress such frameshifts [57]. The modification has been found in human mitochondrial tRNA^Pro^ [196]. The m^1^G_37_ modification occurs in all tRNAs responding to codons beginning with C1 [197]. Mutations in the N1-G_37_ human methyltransferase, TRMT5, are associated with cancers [198]. TRMT5 methylates G_37_ whether there is a G_36_ or C_36_; the bacterial counterpart methylates only G_37_ with an adjacent G_36_. It appears that the modification of the mitochondrial G_37_ is also associated with an oxidative phosphorylation disorder [199].

In summary, the post-transcriptional modification of the position-37 invariant purine takes two forms. The modifications of A_37_ in tRNAs responding to codons beginning with A1 and to some extent also those beginning with U1 improve base stacking for the 3′-stack of the anticodon into the 3′-side of the anticodon loop and into the 3′-side of the anticodon stem. Van der Waals forces, hydrophobic interactions and their solvent-mediated effects of t^6^A_37_ and i^6^A_37_ contribute favorable energetics to stabilizing the base stacking and supporting a conformation advantageous to the Watson–Crick base pairing of the decoding of the first two base pairs. The G_37_ modification to imG_37_ and yW_37_ of tRNA^Phe^ responding to UUU and UUG contributes likewise. In contrast, the less extensive methylation of G_37_ to m^1^G_37_ of tRNAs responding to codons beginning with C1 would not be expected to greatly enhance base stacking. However, purine-37 modifications tend to negate intraloop hydrogen bonding and thus, promote the open loop conformation requisite to the canonical base pairing of the anticodon with the codon. There is little free energy lost in the difference between base hydrogen bonding to one another base or to water [200]. Stacking interactions and solvent displacement drives RNA tertiary structure [201]. Purine-37 modifications maximize anticodon loop stacking and contribute greatly to the desired conformation of the loop structure for mRNA decoding.

### 3.3. Metal Ions and the Modification of the tRNA Anticodon Domain

There is little in the literature expounding upon the importance of metal ions to the contributions of modified nucleoside chemistry and structure to tRNA, certainly in comparison to the extensive literature on the global effect of Mg^2+^ on RNA folding. In some of the earliest papers on modifications and metal ions, the inherent fluorescence of the modification wyosine in yeast tRNA^Phe^ is used to detect the effects of metals on the anticodon structure [202,203]. The fluorescence of the lanthanide metals, such as europium (III), was used to monitor metal binding to specific species of *E. coli* tRNA [204]. These papers confirmed the presence of modest strength, metal binding sites, particularly in the anticodon loop, as seen in crystal structures of tRNAs. In studies of the ASL of yeast tRNA^Phe^ with only the naturally-occurring methylated nucleosides, and m^1^G_37_, the precursor to yW37, the presence of m^5^C and even the deoxynucleoside dm^5^C_40_, modulated cooperative Mg^2+^ binding and enhanced the stability of the ASL [205,206]. Base modifications were found to stabilize or possibly strengthen the binding of Mg^2+^ to *E. coli* tRNA^Val^ [207]. Studies of unmodified yeast tRNA^Phe^ implicated modified nucleoside contributions of chemistry and structure to Mg^2+^ binding and to the correct folding of the fully modified tRNA, thus highlighting the limitations of studying unmodified tRNA [208]. The truncated sequences of mitochondrial tRNAs require modifications in their core regions to facilitate folding into a canonical L-shaped tRNA structure. At least in the folding of human mitochondrial tRNA^Met^ there appears to be an unpredicted Mg^2+^-dependent unfolded state of the 5′-side of the molecule followed by a modification assisted and correct interaction with the 3′-side [209]. The data supports an in vivo model of mitochondrial tRNA transcription and folding in which the truncated DSL remains unfolded until the 5′-side of the molecule is transcribed, and perhaps modified. Modified nucleosides with free amino or acid moieties would be expected to bind metal ions. Studies have shown that metal coordinates to the free acid of the threonine of t^6^A in the *anti*-conformation, and that Mn^2+^ binds the free acid of uridine 5-oxyacetic acetic [210,211]. As would be expected, the binding of Mn^2+^ and Co(NH_3_)_6_^3+^ to the ASL of *E. coli* tRNA^Phe^ neutralizes charge, as would Mg^2+^. Charge neutralization facilitates formation of the ubiquitous U-turn even with an unmodified ASL but when accomplished in the presence of the modified nucleoside, i^6^A_37_, competing ASL conformers are destabilized [212]. It is evident from the study of the modification archaeosine (position 15:,7-formamidino-7-deazaguanosine) that some modifications may, in effect, replace the need for Mg^2+^ to achieve the functional folding of tRNA, particularly with regard to the Levitt base pair, nucleosides at positions 15 and 48 (Figure 3) [62].

### 3.4. Modifications at Position 32

While modifications at position 32 in the tRNA ASL are less commonly considered than those arising at positions 34 and 37, more than 30% of known tRNA species carry a position-32 modification [112]. The nucleoside occupying position 32 is nearly always (>99% of instances) a pyrimidine. Position 32 is located just below the stem region of the anticodon stem and loop and across from the residue at position 38, which is usually unmodified and less strictly conserved [112]. A non-canonical interaction between the position-32 and position-38 nucleosides forms in ASL regions adopting the U-turn structural motif for ribosomal A-site binding, and exhibits a classic bifurcated hydrogen bond between the O2 of the pyrimidine at position 32 (Y_32_) and a hydrogen bond acceptor, typically an amine, in the nucleoside at position 38 (N_38_) in tRNA [213]. This cross-loop base pair interaction significantly affects the ability of the tRNA to engage in codon discrimination, and is also suggested to affect intron splicing and aminoacylation and to modulate affinity of the tRNA for the ribosomal A-site [1,28,214,215]. The cross-loop base pair is stabilized by stacking interactions with nucleosides in the ASL stem, requiring the nucleosides at positions 32 and 38 to adopt the C3′-*endo* sugar pucker found in A-form RNA helices [46,66,92,216,217,218]. The modifications found at position 32, preponderantly 2-thiocytidine or 2′-*O*-methylated pyrimidines, have been shown to stabilize the C3′-*endo* sugar conformation to facilitate base stacking with the ASL stem [112,148].

The 2-thiocytidine (s^2^C) modification at position 32 in *E. coli* tRNA^Arg1^_ICG_ has also been shown to be a direct determinant of wobble decoding efficiency [219]. tRNA^Arg1^_ICG_ and tRNA^Arg2^_ICG_ differ only in their position-32 modification status; unmodified tRNA^Arg2^_ICG_ binds CGC, CGU and CGA arginine codons in agreement with the predictions of the Wobble Hypothesis, but s^2^C_32_-modified tRNA^Arg1^_ICG_ is incapable of wobble binding to CGA [131,219]. The structural basis of the restrictive effect of 2-thiocytidine at position 32 stems from a tendency of the thio modification to contribute to a global, destabilizing dehydration of the ASL under the structural conditions where its conformation has already been perturbed to adapt to a spatially broad adenosine–inosine purine–purine base pair in the wobble position of the anticodon–codon interface [220].

In many cases, tRNA species modified at position 34 also contain modifications at position 32 (67%), position 37 (82%), or both (56%) [112]. Position 32 modifications with position 34 modifications may function in tandem to affect tRNA anticodon loop architecture and function. For instance, it is possible that the thionyl group of the wobble position 34 of 2-thiouridine in *E. coli* tRNA^Lys^_SUU_ may be protected from oxidation through direct or indirect interaction with an *N^6^-*threonylcarbamoyladenosine at position 37, t^6^A_37_ [7,144]. In tRNA^Arg1,2^_ICG_, the 2-thiocytidine and 2-methyladenosine modifications to positions 32 and 37 act to modulate wobble recognition by I_34_ of adenosine in the third position of the anticodon [219]. The pre-structuring of the anticodon domain architecture toward the U-turn structural motif for energetically favorable insertion into the ribosomal A-site, mRNA binding and translocation often requires the simultaneous contributions of several post-transcriptional modifications in the ASL [64,221,222,223]. The U-turn is stabilized by a hydrogen bond between the 2′-OH of U_33_ and, when the residue at position 35 is a purine, its N7 atom; modifications are important in allowing tRNAs with position 35 pyrimidines, such as tRNA^Lys^_UUU_ with mnm^5^s^2^U_34_ and t^6^A_37_, to also adopt U-turns [224]. This motif of interactive contributions of a suite of modifications to positions 32, 34 and 37 of the anticodon stem and loop region seems likely to be in evidence across an array of tRNAs and species.

## 4. Discussion

Modified nucleosides contribute to tRNA folding, structure, and function in numerous ways through their abilities to induce and stabilize the tRNA core, create and pre-structure the most efficient ASL conformation for an accurate response to mRNA codons on the ribosome [225] and act as determinants for recognition by macromolecules due to the unique chemical environment they can provide. Modifications of the ASL in or around the anticodon affect translation. For instance, yeast strains lacking I_34_ at anticodon wobble position 34 are inviable [226], while those lacking m^1^G_37_ and t^6^A_37,_ 3′-adjacent to the anticodon, grow poorly [226,227,228]. Modifications in the core of tRNA affect tRNA stability and folding. Thus, the lack of 1-methyladenosine at position 9 (m^1^A_9_) in human mitochondrial tRNA^Lys^ leads to an alternate elongated structure [31], 2′-*O*-methylation being supportive of the 3′-*endo* nucleoside conformation its absence decreases the probability of the 3′-*endo* conformer [71], and the lack of pseudouridines destabilize helices of the core [229,230]. Some modifications at certain positions affect tRNA identity in protein recognition [224]. In yeast, 2′-O-ribosyladenosine (phosphate), Ar(p), at position 64 in the stem of the TSL is an initiator tRNA^Met^ identity element [231]. The modified nucleosides at positions within the tRNA’s core at the junction of the stems forming the cloverleaf secondary structure tend to negate Watson–Crick, canonical base pairing and have such an important role in the structure and function of the tRNA molecule that mutations at these locations impact human health.

Modified nucleosides with a wide array of chemical moieties enhance the properties of tRNA where both local and global environments can be changed; they pre-structure the molecule both globally and locally for accurate and efficient codon recognition and ribosome binding [20,117,126,130]. The traditional L-shape of tRNA is adopted through highly conserved modifications to the tRNA core enabling a correct global fold to take place. Modified residues at both the D- and T-arm facilitate the three-dimensional interactions through conserved interactions. The D-loop is formed largely due to the increased structural flexibility provided by D, whereas the T-loop often contains highly stabilizing residues such as Ψ and T.

The heightened use of stabilizing nucleosides, such as Ψ and even s^2^T, or on the other hand destabilizing ones such as D, allows organisms to adapt tRNA dynamics to various environments in which life may not be feasible with the limited chemistry provided by only the four major nucleosides. The enhanced C3′-*endo* sugar puckering facilitated by both Ψ and s^2^T stabilize the A-form helix commonly adopted by RNA at 37 °C and enhance the viability of thermophiles at higher temperatures [97]. In contrast, the presence of D induces C2′-*endo* sugar puckering destabilizing the helix, hence increasing structural flexibility due to reduced base stacking. The increased conformational dynamics of the tRNA has enhanced the adaptation of psychrophiles to their inhospitably cold environments [95].

The variety and complexity of modifications found in the ASL and especially at positions 34 and 37 is astounding. The rich variation in chemistry allows for a large number of mechanisms to enable decoding of even thermodynamically weak codons, as well to expand or restrict the recognition of codons. The wobble position is frequently modified regardless of the major base found in the primary sequence, with adenosine and uridine being modified in the majority of cases. With very few exceptions, adenosine is modified to inosine at the wobble position 34 of tRNAs, allowing wider codon recognition through traditional I○A, purine–purine, and I●U/C purine–pyrimidine, Watson–Crick base pairing.

Uridine modifications at position 34 exhibit a rich variety of chemical moieties that can significantly alter the thermodynamics and conformation of the nucleoside. Long carbon chains containing heteroatoms account for properties such as keto–enol and amine–imine tautomerization, ionization, steric hindrance, and increased hydrophobicity. These properties of the long chain modifications often enhance either hydrogen bonding with the cognate codon, enhance base stacking, or restrict binding to non-cognate codons. Additional modifications such as 2′-thiolation further enhance properties such as C3′-*endo* sugar puckering, therefore increasing stacking favorability. The modifications at position-34 often work concomitantly with modifications at position 37.

In the decoding of mRNA, modifications of tRNA’s position-37, 3′-adjacent to the anticodon, stabilize and facilitate formation of the important canonical first base pair in anticodon–codon interaction. The conformation of the ribosome is altered upon tRNA entering the A-site, restricting the first two nucleosides of the codon to forming Watson–Crick base pairs with the tRNA anticodon [232]. Decoding of the first codon base becomes a decisive factor to the accuracy and efficiency of reading the second and third codon bases. tRNA binding the first and second codon bases in a canonical fashion is energetically favored and non-canonical base-paired tRNAs that are less energetically supported are released. Thus, cognate Watson–Crick reading of the first two codon bases leads to efficient and effective binding of the correct tRNAs with few near-cognate mismatches [169]. An accurate first base pair interaction of tRNA with one of the 64 genetic codes would be expected to reduce the potential for a decoding mistake to 1 in 4. Followed by an accurate selection at the second base-pair, there would be a reduction in error to 1 in 16. Formation of the first two canonical base pairs is favored by at least the sum of the hydrogen bond energies of the two base pairs (~−3 kcal/H-bond), ~−12 to −18 kcal. However, an error rate of 1 in 16 is such a large number of mistakes in amino acid incorporation that a protein of median length, 400 amino acids and average molecular size of 44,000 daltons, would have 25 incorrect amino acids. Life as we know it would not be supportable with that error rate. Sustained viability on Earth requires a more energetically favored, structured cycle of tRNA recognition and binding to mRNA codons. Thus, a simplistic purview of the 64 codons of the Universal Genetic Codes and the energy contributions of hydrogen bonds is quite deceiving because many factors greatly improve the mathematical odds of a correct anticodon–codon interaction. In vivo analyses of codon recognition are 10,000-fold more accurate, probably <4 errors in 10^4^ amino acid incorporations [233,234].

Efficient and exact decoding of mRNA is made possible by the rich chemical variation provided by modified nucleosides, especially at the anticodon loop positions 32, 34, and 37. Modifications at positions 32, 34, and 37 facilitate the efficient decoding of pyrimidine rich anticodons by both enabling the formation of the U-turn and enhancing the stability of the anticodon–codon interaction. The half-life of tRNA has been estimated to be from hours to days and the post-transcriptional modifications only add to that time span [235]. Thus, an individual tRNA species responds to its cognate and wobble codons innumerable times during this period. The minimum free energy structure of an unmodified tRNA may not be globally, and specifically at the anticodon loop, structurally fashioned to meet the requirements of the ribosome entry site or the restrictions to translocation. As discussed, the modifications of the tRNA core contribute to the tertiary folding of tRNA into the canonical and functioning three-dimensional shape. For the most part, modifications in the core, methylations in particular, negate Watson–Crick base pairing resulting in a three-dimensional structure with non-canonical base pairs and base triples of varying stability [236,237]. The modifications of the anticodon stem and loop enable some 40 tRNAs to read the 61 amino acid codons, and yet retain a common conformation and dynamic acceptable to translation. Thus, with few exceptions, every time an unmodified or under modified tRNA responds to its codon, it may have to undergo an investment of energy and time in restructuring to meet ribosome requirements. Modifications engineer and pre-structure the tRNA architecture and alter its dynamics in the direction of ribosome acceptance. Therefore, the modifications discussed in this review enable the correct folding, and accurate and efficient functioning of tRNA in its central role in biology.

## Supplementary Materials

Supplementary File 1

## References

[1] Agris P.F. (2004). Decoding the genome: A modified view. Nucleic Acids Res..

[2] Agris P.F. (2015). The importance of being modified: An unrealized code to RNA structure and function. RNA.

[3] Zhao B.S., Roundtree I.A., He C. (2017). Post-transcriptional gene regulation by mRNA modifications. Nat. Rev. Mol. Cell Biol..

[4] Duechler M., Leszczyńska G., Sochacka E., Nawrot B. (2016). Nucleoside modifications in the regulation of gene expression: focus on tRNA. Cell. Mol. Life Sci..

[5] Cantara W.A., Crain P.F., Rozenski J., McCloskey J.A., Harris K.A., Zhang X., Vendeix F.A., Fabris D., Agris P.F. (2011). The RNA modification database, RNAMDB: 2011 update. Nucleic Acids Res..

[6] Machnicka M.A., Milanowska K., Osman Oglou O., Purta E., Kurkowska M., Olchowik A., Januszewski W., Kalinowski S., Dunin-Horkawicz S., Rother K.M. (2013). MODOMICS: A database of RNA modification pathways--2013 update. Nucleic Acids Res..

[7] Agris P.F. (1996). The importance of being modified: Roles of modified nucleosides and Mg^2+^ in RNA structure and function. Prog. Nucleic Acid Res. Mol. Biol..

[8] Schmidt P.G., Sierzputowska-Gracz H., Agris P.F. (1987). Internal motions in yeast phenylalanine transfer RNA from ^13^C NMR relaxation rates of modified base methyl groups: A model-free approach. Biochemistry.

[9] Agris P.F. (1991). Wobble position modified nucleosides evolved to select transfer RNA codon recognition: A modified-wobble hypothesis. Biochimie.

[10] Björk G.R., Söll D., RajBhandary U. (1995). Chapter 11: Biosynthesis and Function of Modified Nucleosides. tRNA: Structure, Biosynthesis, and Function.

[11] Suzuki T., Suzuki T. (2014). A complete landscape of post-transcriptional modifications in mammalian mitochondrial tRNAs. Nucleic Acids Res..

[12] Björk G., Hagervall T. (2014). Transfer RNA modification: Presence, synthesis, and function. EcoSal Plus.

[13] Jackman J.E., Alfonzo J.D. (2013). Transfer RNA modifications: Nature’s combinatorial chemistry playground. Wiley Interdiscip. Rev. RNA.

[14] Helm M., Alfonzo J.D. (2014). Posttranscriptional RNA modifications: Playing metabolic games in a cell’s chemical Legoland. Chem. Biol..

[15] Carell T., Brandmayr C., Hienzsch A., Muller M., Pearson D., Reiter V., Thoma I., Thumbs P., Wagner M. (2012). Structure and function of noncanonical nucleobases. Angew. Chem. Int. Ed. Engl..

[16] Basavappa R., Sigler P.B. (1991). The 3 Å crystal structure of yeast initiator tRNA: Functional implications in initiator/elongator discrimination. EMBO J..

[17] Lewis C.J., Pan T., Kalsotra A. (2017). RNA modifications and structures cooperate to guide RNA-protein interactions. Nat. Rev. Mol. Cell Biol..

[18] Zhang J., Ferre-D’Amare A.R. (2013). Co-crystal structure of a T-box riboswitch stem I domain in complex with its cognate tRNA. Nature.

[19] Machnicka M.A., Olchowik A., Grosjean H., Bujnicki J.M. (2014). Distribution and frequencies of post-transcriptional modifications in tRNAs. RNA Biol..

[20] Agris P.F., Vendeix F.A., Graham W.D. (2007). tRNA’s wobble decoding of the genome: 40 years of modification. J. Mol. Biol..

[21] Hamann C.S., Hou Y.-M. (2000). Probing a tRNA core that contributes to aminoacylation^1^. J. Mol. Biol..

[22] Grosjean H., Edqvist J., Stråby K.B., Giegé R. (1996). Enzymatic formation of modified nucleosides in tRNA: Dependence on tRNA Architecture. J. Mol. Biol..

[23] Dirheimer G., Keith G., Dumas P., Westhof E., Söll D., RajBhandary U.L. (1995). tRNA: Structure, Biosynthesis, and Function.

[24] Giegé R., Puglisi J.D., Florentz C., Waldo E.C., Kivie M. (1993). tRNA Structure and Aminoacylation Efficiency. Progress in Nucleic Acid Research and Molecular Biology.

[25] Christian T., Lipman R.S., Evilia C., Hou Y.M. (2000). Alternative design of a tRNA core for aminoacylation. J. Mol. Biol..

[26] Klug A., Ladner J., Robertus J.D. (1974). The structural geometry of co-ordinated base changes in transfer RNA. J. Mol. Biol..

[27] Sprinzl M., Horn C., Brown M., Ioudovitch A., Steinberg S. (1998). Compilation of tRNA sequences and sequences of tRNA genes. Nucleic Acids Res..

[28] Giegé R., Sissler M., Florentz C. (1998). Universal rules and idiosyncratic features in tRNA identity. Nucleic Acids Res..

[29] Sampson J.R., DiRenzo A.B., Behlen L.S., Uhlenbeck O.C. (1989). Nucleotides in yeast tRNA^Phe^ required for the specific recognition by its cognate synthetase. Science.

[30] McClain W.H., Foss K. (1988). Nucleotides that contribute to the identity of *Escherichia coli* tRNA^Phe^. J. Mol. Biol..

[31] Helm M., Giegé R., Florentz C. (1999). A Watson–Crick base-pair-disrupting methyl group (m^1^A9) is sufficient for cloverleaf folding of human mitochondrial tRNA^Lys^. Biochemistry.

[32] Sakurai M., Ohtsuki T., Watanabe K. (2005). Modification at position 9 with 1-methyladenosine is crucial for structure and function of nematode mitochondrial tRNAs lacking the entire T-arm. Nucleic Acids Res..

[33] Vilardo E., Nachbagauer C., Buzet A., Taschner A., Holzmann J., Rossmanith W. (2012). A subcomplex of human mitochondrial RNase P is a bifunctional methyltransferase–extensive moonlighting in mitochondrial tRNA biogenesis. Nucleic Acids Res..

[34] Oerum S., Dégut C., Barraud P., Tisné C. (2017). m^1^A post-transcriptional modification in tRNAs. Biomolecules.

[35] Gillis D., Krishnamohan A., Yaacov B., Shaag A., Jackman J.E., Elpeleg O. (2014). TRMT10A dysfunction is associated with abnormalities in glucose homeostasis, short stature and microcephaly. J. Med. Genet..

[36] Voigts-Hoffmann F., Hengesbach M., Kobitski A.Y., van Aerschot A., Herdewijn P., Nienhaus G.U., Helm M. (2007). A methyl group controls conformational equilibrium in human mitochondrial tRNA^Lys^. J. Am. Chem. Soc..

[37] Helm M., Attardi G. (2004). Nuclear control of cloverleaf structure of human mitochondrial tRNA^Lys^. J. Mol. Biol..

[38] Sissler M., Helm M., Frugier M., Giege R., Florentz C. (2004). Aminoacylation properties of pathology-related human mitochondrial tRNA^Lys^ variants. RNA.

[39] Holzmann J., Frank P., Löffler E., Bennett K.L., Gerner C., Rossmanith W. (2008). RNase P without RNA: Identification and functional reconstitution of the human mitochondrial tRNA processing enzyme. Cell.

[40] Kobitski A.Y., Hengesbach M., Helm M., Nienhaus G.U. (2008). Sculpting an RNA conformational energy landscape by a methyl group modification—A single-molecule FRET study. Angew. Chem. Int. Ed..

[41] Motorin Y., Helm M. (2010). tRNA stabilization by modified nucleotides. Biochemistry.

[42] Nobles K.N., Yarian C.S., Liu G., Guenther R.H., Agris P.F. (2002). Highly conserved modified nucleosides influence Mg^2+^-dependent tRNA folding. Nucleic Acids Res..

[43] Jackman J.E., Montange R.K., Malik H.S., Phizicky E.M. (2003). Identification of the yeast gene encoding the tRNA m^1^G methyltransferase responsible for modification at position 9. RNA.

[44] Saenger W. (1984). Intercalation. Principles of Nucleic Acid Structure.

[45] Noon K.R., Guymon R., Crain P.F., McCloskey J.A., Thomm M., Lim J., Cavicchioli R. (2003). Influence of temperature on tRNA modification in archaea: *Methanococcoides burtonii* (optimum growth temperature [T_opt_], 23 °C) and *Stetteria hydrogenophila* (T_opt_, 95 °C). J. Bacteriol..

[46] Shi H., Moore P.B. (2000). The crystal structure of yeast phenylalanine tRNA at 1.93 A resolution: A classic structure revisited. RNA.

[47] Hayase Y., Jahn M., Rogers M.J., Sylvers L.A., Koizumi M., Inoue H., Ohtsuka E., Söll D. (1992). Recognition of bases in *Escherichia coli* tRNA^Gln^ by glutaminyl-tRNA synthetase: A complete identity set. EMBO J..

[48] Sprinzl M., Hartmann T., Weber J., Blank J., Zeidler R. (1989). Compilation of tRNA sequences and sequences of tRNA genes. Nucleic Acids Res..

[49] Edqvist J., Grosjean H., Straby K.B. (1992). Identity elements for N^2^-dimethylation of guanosine-26 in yeast tRNAs. Nucleic Acids Res..

[50] Arimbasseri A.G., Blewett N.H., Iben J.R., Lamichhane T.N., Cherkasova V., Hafner M., Maraia R.J. (2015). RNA polymerase III output is functionally linked to tRNA dimethyl-G26 modification. PLoS Genet..

[51] Hori H. (2014). Methylated nucleosides in tRNA and tRNA methyltransferases. Front. Genet..

[52] Burgess A.L., David R., Searle I.R. (2015). Conservation of tRNA and rRNA 5-methylcytosine in the kingdom Plantae. BMC Plant Biol..

[53] Tuorto F., Liebers R., Musch T., Schaefer M., Hofmann S., Kellner S., Frye M., Helm M., Stoecklin G., Lyko F. (2012). RNA cytosine methylation by Dnmt2 and NSun2 promotes tRNA stability and protein synthesis. Nat. Struct. Mol. Biol..

[54] Motorin Y., Grosjean H. (1999). Multisite-specific tRNA:m^5^C-methyltransferase (Trm4) in yeast *Saccharomyces cerevisiae*: Identification of the gene and substrate specificity of the enzyme. RNA.

[55] Blanco S., Kurowski A., Nichols J., Watt F.M., Benitah S.A., Frye M. (2011). The RNA-methyltransferase Misu (NSun2) poises epidermal stem cells to differentiate. PLoS Genet..

[56] Blanco S., Dietmann S., Flores J.V., Hussain S., Kutter C., Humphreys P., Lukk M., Lombard P., Treps L., Popis M. (2014). Aberrant methylation of tRNAs links cellular stress to neuro-developmental disorders. EMBO J..

[57] Hou Y.M., Gamper H., Yang W. (2015). Post-transcriptional modifications to tRNA—A response to the genetic code degeneracy. RNA.

[58] Auxilien S., Guerineau V., Szweykowska-Kulinska Z., Golinelli-Pimpaneau B. (2012). The human tRNA m^5^C methyltransferase Misu is multisite-specific. RNA Biol..

[59] Militello K.T., Chen L.M., Ackerman S.E., Mandarano A.H., Valentine E.L. (2014). A map of 5-methylcytosine residues in *Trypanosoma brucei* tRNA revealed by sodium bisulfite sequencing. Mol. Biochem. Parasitol..

[60] Levitt M. (1969). Detailed molecular model for transfer ribonucleic acid. Nature.

[61] Burkhard M.E., Turner D.H., Tinoco I., Gesteland R.F., Cech T.R., Atkins J.F. (1999). The interactions that shape RNA structure. The RNA World.

[62] Oliva R., Tramontano A., Cavallo L. (2007). Mg^2+^ binding and archaeosine modification stabilize the G15–C48 Levitt base pair in tRNAs. RNA.

[63] Gregson J.M., Crain P.F., Edmonds C.G., Gupta R., Hashizume T., Phillipson D.W., McCloskey J.A. (1993). Structure of the archaeal transfer RNA nucleoside G*-15 (2-amino-4,7-dihydro-4-oxo-7-*β*-d-ribofuranosyl-1*H*-pyrrolo[2,3-*d*]pyrimidine-5-carboximidamide (archaeosine)). J. Biol. Chem..

[64] Quigley G.J., Rich A. (1976). Structural domains of transfer RNA molecules. Science.

[65] Sherlin L.D., Bullock T.L., Newberry K.J., Lipman R.S., Hou Y.M., Beijer B., Sproat B.S., Perona J.J. (2000). Influence of transfer RNA tertiary structure on aminoacylation efficiency by glutaminyl and cysteinyl-tRNA synthetases. J. Mol. Biol..

[66] Nissen P., Thirup S., Kjeldgaard M., Nyborg J. (1999). The crystal structure of Cys-tRNA^Cys^-EF-Tu-GDPNP reveals general and specific features in the ternary complex and in tRNA. Structure.

[67] Hou Y.M., Westhof E., Giege R. (1993). An unusual RNA tertiary interaction has a role for the specific aminoacylation of a transfer RNA. Proc. Natl. Acad. Sci. USA.

[68] Hou Y.M., Sterner T., Jansen M. (1995). Permutation of a pair of tertiary nucleotides in a transfer RNA. Biochemistry.

[69] Cusack S., Yaremchuk A., Tukalo M. (1996). The crystal structure of the ternary complex of *T.thermophilus* seryl-tRNA synthetase with tRNA^Ser^ and a seryl-adenylate analogue reveals a conformational switch in the active site. EMBO J..

[70] Kawai G., Ue H., Yasuda M., Sakamoto K., Hashizume T., McCloskey J.A., Miyazawa T., Yokoyama S. (1991). Relation between functions and conformational characteristics of modified nucleosides found in tRNAs. Nucleic Acids Symp. Ser..

[71] Kawai G., Yamamoto Y., Kamimura T., Masegi T., Sekine M., Hata T., Iimori T., Watanabe T., Miyazawa T., Yokoyama S. (1992). Conformational rigidity of specific pyrimidine residues in tRNA arises from posttranscriptional modifications that enhance steric interaction between the base and the 2’-hydroxyl group. Biochemistry.

[72] Agris P.F., Koh H., Soll D. (1973). The effect of growth temperatures on the in vivo ribose methylation of *Bacillus stearothermophilus* transfer RNA. Arch. Biochem. Biophys..

[73] Kumagai I., Watanabe K., Oshima T. (1980). Thermally induced biosynthesis of 2’-*O*-methylguanosine in tRNA from an extreme thermophile, *Thermus thermophilus* HB27. Proc. Natl. Acad. Sci. USA.

[74] Droogmans L., Roovers M., Bujnicki J.M., Tricot C., Hartsch T., Stalon V., Grosjean H. (2003). Cloning and characterization of tRNA (m^1^A_58_) methyltransferase (TrmI) from *Thermus thermophilus* HB27, a protein required for cell growth at extreme temperatures. Nucleic Acids Res..

[75] Tomikawa C., Yokogawa T., Kanai T., Hori H. (2010). *N*^7^-Methylguanine at position 46 (m^7^G_46_) in tRNA from *Thermus thermophilus* is required for cell viability at high temperatures through a tRNA modification network. Nucleic Acids Res..

[76] Shaheen R., Abdel-Salam G.M.H., Guy M.P., Alomar R., Abdel-Hamid M.S., Afifi H.H., Ismail S.I., Emam B.A., Phizicky E.M., Alkuraya F.S. (2015). Mutation in *WDR4* impairs tRNA m^7^G_46_ methylation and causes a distinct form of microcephalic primordial dwarfism. Genome Biol..

[77] Anderson J., Phan L., Cuesta R., Carlson B.A., Pak M., Asano K., Bjork G.R., Tamame M., Hinnebusch A.G. (1998). The essential Gcd10p-Gcd14p nuclear complex is required for 1-methyladenosine modification and maturation of initiator methionyl-tRNA. Genes Dev..

[78] Saikia M., Fu Y., Pavon-Eternod M., He C., Pan T. (2010). Genome-wide analysis of *N*^1^-methyl-adenosine modification in human tRNAs. RNA.

[79] Oliva R., Cavallo L., Tramontano A. (2006). Accurate energies of hydrogen bonded nucleic acid base pairs and triplets in tRNA tertiary interactions. Nucleic Acids Res..

[80] Morla-Folch J., Xie H.N., Alvarez-Puebla R.A., Guerrini L. (2016). Fast optical chemical and structural classification of RNA. ACS Nano.

[81] Davanloo P., Sprinzl M., Watanabe K., Albani M., Kersten H. (1979). Role of ribothymidine in the thermal stability of transfer RNA as monitored by proton magnetic resonance. Nucleic Acids Res..

[82] Dalluge J.J., Hashizume T., Sopchik A.E., McCloskey J.A., Davis D.R. (1996). Conformational flexibility in RNA: The role of dihydrouridine. Nucleic Acids Res..

[83] Dyubankova N., Sochacka E., Kraszewska K., Nawrot B., Herdewijn P., Lescrinier E. (2015). Contribution of dihydrouridine in folding of the D-arm in tRNA. Org. Biomol. Chem..

[84] Nawrot B., Malkiewicz A., Smith W.S., Sierzputowska-Gracz H., Agris P.F. (1995). RNA modified uridines VII: Chemical synthesis and initial analysis of tRNA D-Loop oligomers with tandem modified uridines. Nucleosides Nucleotides.

[85] Stuart J.W., Basti M.M., Smith W.S., Forrest B., Guenther R., Sierzputowska-Gracz H., Nawrot B., Malkiewicz A., Agris P.F. (1996). Structure of the trinucleotide D-acp^3^U-A with coordinated Mg^2+^ demonstrates that modified nucleosides contribute to regional conformations of RNA. Nucleosides Nucleotides.

[86] Suck D., Saenger W., Zechmeister K. (1972). Molecular and crystal structure of the tRNA minor constituent dihydrouridine. Acta Crystallogr. Sect. B: Struct. Sci..

[87] Egert E., Lindner H.J., Hillen W., Boehm M.C. (1980). Influence of substituents at the 5 position on the structure of uridine. J. Am. Chem. Soc..

[88] Uhl W., Reiner J., Gassen H.G. (1983). On the conformation of 5-substituted uridines as studied by proton magnetic resonance. Nucleic Acids Res..

[89] Sundaralingam M., Rao S.T., Abola J. (1971). Stereochemistry of nucleic acids and their constituents. XXIII. Crystal and molecular structure of dihydrouridine “hemihydrate”, a rare nucleoside with a saturated base occurring in the dihydrouridine loop of transfer ribonucleic acids. J. Am. Chem. Soc..

[90] Deslauriers R., Smith I.C.P. (1973). A comparison of the conformations of uridine, *β*-pseudouridine, and dihydrouridine in dimethyl sulfoxide and water. A ^1^H nuclear magnetic resonance study. Can. J. Chem..

[91] Sipa K., Sochacka E., Kazmierczak-Baranska J., Maszewska M., Janicka M., Nowak G., Nawrot B. (2007). Effect of base modifications on structure, thermodynamic stability, and gene silencing activity of short interfering RNA. RNA.

[92] Westhof E., Sundaralingam M. (1986). Restrained refinement of the monoclinic form of yeast phenylalanine transfer RNA. Temperature factors and dynamics, coordinated waters, and base-pair propeller twist angles. Biochemistry.

[93] Westhof E., Dumas P., Moras D. (1985). Crystallographic refinement of yeast aspartic acid transfer RNA. J. Mol. Biol..

[94] Morita R.Y. (1975). Psychrophilic bacteria. Bacteriol. Rev..

[95] Dalluge J.J., Hamamoto T., Horikoshi K., Morita R.Y., Stetter K.O., McCloskey J.A. (1997). Posttranscriptional modification of tRNA in psychrophilic bacteria. J. Bacteriol..

[96] Watanabe K., Oshima T., Iijima K., Yamaizumi Z., Nishimura S. (1980). Purification and thermal stability of several amino acid-specific tRNAs from an extreme thermophile, *Thermus thermophilus* HB8. J. Biochem..

[97] Yamamoto Y., Yokoyama S., Miyazawa T., Watanabe K., Higuchi S. (1983). NMR analyses on the molecular mechanism of the conformational rigidity of 2-thioribothymidine, a modified nucleoside in extreme thermophile tRNAs. FEBS Lett..

[98] Horie N., Hara-Yokoyama M., Yokoyama S., Watanabe K., Kuchino Y., Nishimura S., Miyazawa T. (1985). Two tRNA^Ile1^ species from an extreme thermophile, *Thermus thermophilus* HB8: Effect of 2-thiolation of ribothymidine on the thermostability of tRNA. Biochemistry.

[99] Carlile T.M., Rojas-Duran M.F., Zinshteyn B., Shin H., Bartoli K.M., Gilbert W.V. (2014). Pseudouridine profiling reveals regulated mRNA pseudouridylation in yeast and human cells. Nature.

[100] Hudson G.A., Bloomingdale R.J., Znosko B.M. (2013). Thermodynamic contribution and nearest-neighbor parameters of pseudouridine-adenosine base pairs in oligoribonucleotides. RNA.

[101] Charette M., Gray M.W. (2000). Pseudouridine in RNA: What, where, how, and why. IUBMB Life.

[102] Gilbert W.V., Bell T.A., Schaening C. (2016). Messenger RNA modifications: Form, distribution, and function. Science.

[103] Hamma T., Ferre-D’Amare A.R. (2006). Pseudouridine synthases. Chem. Biol..

[104] Veerareddygari G.R., Singh S.K., Mueller E.G. (2016). The pseudouridine synthases proceed through a glycal intermediate. J. Am. Chem. Soc..

[105] Spenkuch F., Motorin Y., Helm M. (2014). Pseudouridine: Still mysterious, but never a fake (uridine)!. RNA Biol..

[106] Davis D.R. (1995). Stabilization of RNA stacking by pseudouridine. Nucleic Acids Res..

[107] Xu Y., Vanommeslaeghe K., Aleksandrov A., MacKerell A.D., Nilsson L. (2016). Additive CHARMM force field for naturally occurring modified ribonucleotides. J. Comput. Chem..

[108] Nanda R., Tewari R., Govil G., Smith I.C. (1974). The conformation of *β*-pseudouridine about the glycosidic bond as studied by ^1^H homonuclear overhauser measurements and molecular orbital calculations. Can. J. Chem..

[109] Neumann J.M., Bernassau J.M., Gueron M., Tran-Dinh S. (1980). Comparative conformations of uridine and pseudouridine and their derivatives. Eur. J. Biochem..

[110] Yarian C.S., Basti M.M., Cain R.J., Ansari G., Guenther R.H., Sochacka E., Czerwinska G., Malkiewicz A., Agris P.F. (1999). Structural and functional roles of the N1- and N3-protons of Ψ at tRNA’s position 39. Nucleic Acids Res..

[111] Arluison V., Buckle M., Grosjean H. (1999). Pseudouridine synthetase pus1 of *Saccharomyces cerevisiae*: Kinetic characterisation, tRNA structural requirement and real-time analysis of its complex with tRNA^1^. J. Mol. Biol..

[112] Juhling F., Morl M., Hartmann R.K., Sprinzl M., Stadler P.F., Putz J. (2009). tRNAdb 2009: Compilation of tRNA sequences and tRNA genes. Nucleic Acids Res..

[113] Lusic H., Gustilo E.M., Vendeix F.A., Kaiser R., Delaney M.O., Graham W.D., Moye V.A., Cantara W.A., Agris P.F., Deiters A. (2008). Synthesis and investigation of the 5-formylcytidine modified, anticodon stem and loop of the human mitochondrial tRNA^Met^. Nucleic Acids Res..

[114] Stuart J.W., Koshlap K.M., Guenther R., Agris P.F. (2003). Naturally-occurring modification restricts the anticodon domain conformational space of tRNA^Phe^. J. Mol. Biol..

[115] Yarian C., Marszalek M., Sochacka E., Malkiewicz A., Guenther R., Miskiewicz A., Agris P.F. (2000). Modified nucleoside dependent Watson-Crick and wobble codon binding by tRNA^LysUUU^ species. Biochemistry.

[116] Davis D.R., Durant P.C. (1999). Nucleoside modifications affect the structure and stability of the anticodon of tRNA^Lys,3^. Nucleosides Nucleotides.

[117] Denmon A.P., Wang J., Nikonowicz E.P. (2011). Conformation effects of base modification on the anticodon stem-loop of *Bacillus subtilis* tRNA^Tyr^. J. Mol. Biol..

[118] Cabello-Villegas J., Winkler M.E., Nikonowicz E.P. (2002). Solution conformations of unmodified and A_37_N^6^-dimethylallyl modified anticodon stem-loops of *Escherichia coli* tRNA^Phe^. J. Mol. Biol..

[119] Yarian C., Townsend H., Czestkowski W., Sochacka E., Malkiewicz A.J., Guenther R., Miskiewicz A., Agris P.F. (2002). Accurate translation of the genetic code depends on tRNA modified nucleosides. J. Biol. Chem..

[120] Kurata S., Weixlbaumer A., Ohtsuki T., Shimazaki T., Wada T., Kirino Y., Takai K., Watanabe K., Ramakrishnan V., Suzuki T. (2008). Modified uridines with C5-methylene substituents at the first position of the tRNA anticodon stabilize U.G wobble pairing during decoding. J. Biol. Chem..

[121] Phelps S.S., Malkiewicz A., Agris P.F., Joseph S. (2004). Modified nucleotides in tRNA^Lys^ and tRNA^Val^ are important for translocation. J. Mol. Biol..

[122] Ashraf S.S., Sochacka E., Cain R., Guenther R., Malkiewicz A., Agris P.F. (1999). Single atom modification (O→S) of tRNA confers ribosome binding. RNA.

[123] Li J., Esberg B., Curran J.F., Bjork G.R. (1997). Three modified nucleosides present in the anticodon stem and loop influence the in vivo aa-tRNA selection in a tRNA-dependent manner. J. Mol. Biol..

[124] Chiari Y., Dion K., Colborn J., Parmakelis A., Powell J.R. (2010). On the possible role of tRNA base modifications in the evolution of codon usage: Queuosine and *Drosophila*. J. Mol. Evol..

[125] Ninio J. (2006). Multiple stages in codon-anticodon recognition: Double-trigger mechanisms and geometric constraints. Biochimie.

[126] Gustilo E.M., Vendeix F.A., Agris P.F. (2008). tRNA’s modifications bring order to gene expression. Curr. Opin. Microbiol..

[127] Urbonavicius J., Qian Q., Durand J.M., Hagervall T.G., Bjork G.R. (2001). Improvement of reading frame maintenance is a common function for several tRNA modifications. EMBO J..

[128] Bjork G.R., Durand J.M., Hagervall T.G., Leipuviene R., Lundgren H.K., Nilsson K., Chen P., Qian Q., Urbonavicius J. (1999). Transfer RNA modification: Influence on translational frameshifting and metabolism. FEBS Lett..

[129] Brierley I., Meredith M.R., Bloys A.J., Hagervall T.G. (1997). Expression of a coronavirus ribosomal frameshift signal in *Escherichia coli*: Influence of tRNA anticodon modification on frameshifting. J. Mol. Biol..

[130] Agris P.F. (2008). Bringing order to translation: The contributions of transfer RNA anticodon-domain modifications. EMBO Rep..

[131] Crick F.H. (1966). Codon–anticodon pairing: The wobble hypothesis. J. Mol. Biol..

[132] Lagerkvist U. (1978). “Two out of three”: An alternative method for codon reading. Proc. Natl. Acad. Sci. USA.

[133] Lagerkvist U. (1981). Unorthodox codon reading and the evolution of the genetic code. Cell.

[134] Wakasugi T., Ohme M., Shinozaki K., Sugiura M. (1986). Structures of tobacco chloroplast genes for tRNA^Ile^ (CAU), tRNA^Leu^ (CAA), tRNA^Cys^ (GCA), tRNA^Ser^ (UGA) and tRNA^Thr^ (GGU): A compilation of tRNA genes from tobacco chloroplasts. Plant Mol. Biol..

[135] Rogalski M., Karcher D., Bock R. (2008). Superwobbling facilitates translation with reduced tRNA sets. Nat. Struct. Mol. Biol..

[136] Weixlbaumer A., Murphy F.V.t., Dziergowska A., Malkiewicz A., Vendeix F.A., Agris P.F., Ramakrishnan V. (2007). Mechanism for expanding the decoding capacity of transfer RNAs by modification of uridines. Nat. Struct. Mol. Biol..

[137] Ishikura H., Yamada Y., Nishimura S. (1971). Structure of serine tRNA from *Escherichia coli*. I. Purification of serine tRNA’s with different codon responses. Biochim. Biophys. Acta.

[138] Mitra S.K., Lustig F., Akesson B., Axberg T., Elias P., Lagerkvist U. (1979). Relative efficiency of anticodons in reading the valine codons during protein synthesis in vitro. J. Biol. Chem..

[139] Samuelsson T., Elias P., Lustig F., Axberg T., Folsch G., Akesson B., Lagerkvist U. (1980). Aberrations of the classic codon reading scheme during protein synthesis in vitro. J. Biol. Chem..

[140] Sakamoto K., Kawai G., Niimi T., Satoh T., Sekine M., Yamaizumi Z., Nishimura S., Miyazawa T., Yokoyama S. (1993). A modified uridine in the first position of the anticodon of a minor species of arginine tRNA, the *argU* gene product, from *Escherichia coli*. Eur. J. Biochem..

[141] Spanjaard R.A., Chen K., Walker J.R., van Duin J. (1990). Frameshift suppression at tandem AGA and AGG codons by cloned tRNA genes: Assigning a codon to *argU* tRNA and T4 tRNA^Arg^. Nucleic Acids Res..

[142] Johansson M.J., Esberg A., Huang B., Bjork G.R., Bystrom A.S. (2008). Eukaryotic wobble uridine modifications promote a functionally redundant decoding system. Mol. Cell. Biol..

[143] Takai K., Yokoyama S. (2003). Roles of 5-substituents of tRNA wobble uridines in the recognition of purine-ending codons. Nucleic Acids Res..

[144] Agris P.F., Guenther R., Ingram P.C., Basti M.M., Stuart J.W., Sochacka E., Malkiewicz A. (1997). Unconventional structure of tRNA^LysSUU^ anticodon explains tRNA’s role in bacterial and mammalian ribosomal frameshifting and primer selection by HIV-1. RNA.

[145] Agris P.F., Soll D., Seno T. (1973). Biological function of 2-thiouridine in *Escherichia coli* glutamic acid transfer ribonucleic acid. Biochemistry.

[146] Yokoyama S., Watanabe T., Murao K., Ishikura H., Yamaizumi Z., Nishimura S., Miyazawa T. (1985). Molecular mechanism of codon recognition by tRNA species with modified uridine in the first position of the anticodon. Proc. Natl. Acad. Sci. USA.

[147] Lustig F., Elias P., Axberg T., Samuelsson T., Tittawella I., Lagerkvist U. (1981). Codon reading and translational error. Reading of the glutamine and lysine codons during protein synthesis in vitro. J. Biol. Chem..

[148] Bilbille Y., Vendeix F.A., Guenther R., Malkiewicz A., Ariza X., Vilarrasa J., Agris P.F. (2009). The structure of the human tRNA^Lys3^ anticodon bound to the HIV genome is stabilized by modified nucleosides and adjacent mismatch base pairs. Nucleic Acids Res..

[149] Yokoyama S., Nishimura S., Schimmel P.R., Söll D., RajBhandary U.L. (1995). Modified nucleosides and codon recognition. tRNA: Structure, Biosynthesis and Function.

[150] Yasukawa T., Suzuki T., Ishii N., Ueda T., Ohta S., Watanabe K. (2000). Defect in modification at the anticodon wobble nucleotide of mitochondrial tRNA^Lys^ with the MERRF encephalomyopathy pathogenic mutation. FEBS Lett..

[151] Kirino Y., Suzuki T. (2005). Human mitochondrial diseases associated with tRNA wobble modification deficiency. RNA Biol..

[152] Yasukawa T., Kirino Y., Ishii N., Holt I.J., Jacobs H.T., Makifuchi T., Fukuhara N., Ohta S., Suzuki T., Watanabe K. (2005). Wobble modification deficiency in mutant tRNAs in patients with mitochondrial diseases. FEBS Lett..

[153] Kamble A.S., Kumbhar B.V., Sambhare S.B., Bavi R.S., Sonawane K.D. (2015). Conformational preferences of modified nucleoside 5-taurinomethyluridine, τm5U occur at ‘wobble’ 34th position in the anticodon loop of tRNA. Cell Biochem. Biophys..

[154] Wang R., Vangaveti S., Ranganathan S.V., Basanta-Sanchez M., Haruehanroengra P., Chen A., Sheng J. (2016). Synthesis, base pairing and structure studies of geranylated RNA. Nucleic Acids Res..

[155] Wang R., Ranganathan S.V., Basanta-Sanchez M., Shen F., Chen A., Sheng J. (2015). Synthesis and base pairing studies of geranylated 2-thiothymidine, a natural variant of thymidine. Chem. Commun..

[156] Sierant M., Leszczynska G., Sadowska K., Dziergowska A., Rozanski M., Sochacka E., Nawrot B. (2016). *S*-Geranyl-2-thiouridine wobble nucleosides of bacterial tRNAs; chemical and enzymatic synthesis of *S*-geranylated-RNAs and their physicochemical characterization. Nucleic Acids Res..

[157] Andachi Y., Yamao F., Iwami M., Muto A., Osawa S. (1987). Occurrence of unmodified adenine and uracil at the first position of anticodon in threonine tRNAs in *Mycoplasma capricolum*. Proc. Natl. Acad. Sci. USA.

[158] Andachi Y., Yamao F., Muto A., Osawa S. (1989). Codon recognition patterns as deduced from sequences of the complete set of transfer RNA species in *Mycoplasma capricolum*. Resemblance to mitochondria. J. Mol. Biol..

[159] Guindy Y.S., Samuelsson T., Johansen T.I. (1989). Unconventional codon reading by *Mycoplasma mycoides* tRNAs as revealed by partial sequence analysis. Biochem. J..

[160] Samuelsson T., Guindy Y.S., Lustig F., Boren T., Lagerkvist U. (1987). Apparent lack of discrimination in the reading of certain codons in *Mycoplasma mycoides*. Proc. Natl. Acad. Sci. USA.

[161] Sibler A.P., Dirheimer G., Martin R.P. (1986). Codon reading patterns in *Saccharomyces cerevisiae* mitochondria based on sequences of mitochondrial tRNAs. FEBS Lett..

[162] Watanabe Y., Tsurui H., Ueda T., Furusihima-Shimogawara R., Takamiya S., Kita K., Nishikawa K., Watanabe K. (1997). Primary sequence of mitochondrial tRNA^Arg^ of a nematode *Ascaris suum*: occurrence of unmodified adenosine at the first position of the anticodon. Biochim. Biophys. Acta.

[163] Murphy F.V.t., Ramakrishnan V. (2004). Structure of a purine-purine wobble base pair in the decoding center of the ribosome. Nat. Struct. Mol. Biol..

[164] Deffit S.N., Hundley H.A. (2016). To edit or not to edit: Regulation of ADAR editing specificity and efficiency. Wiley Interdiscip. Rev. RNA.

[165] Zinshteyn B., Nishikura K. (2009). Adenosine-to-inosine RNA editing. Wiley Interdiscip. Rev. Syst. Biol. Med..

[166] Mannion N., Arieti F., Gallo A., Keegan L.P., O’Connell M.A. (2015). New insights into the biological role of mammalian ADARs; the RNA editing proteins. Biomolecules.

[167] Vendeix F.A., Murphy F.V.t., Cantara W.A., Leszczynska G., Gustilo E.M., Sproat B., Malkiewicz A., Agris P.F. (2012). Human tRNA^Lys3UUU^ is pre-structured by natural modifications for cognate and wobble codon binding through keto-enol tautomerism. J. Mol. Biol..

[168] Rozov A., Demeshkina N., Khusainov I., Westhof E., Yusupov M., Yusupova G. (2016). Novel base-pairing interactions at the tRNA wobble position crucial for accurate reading of the genetic code. Nat. Commun..

[169] Rozov A., Westhof E., Yusupov M., Yusupova G. (2016). The ribosome prohibits the G*U wobble geometry at the first position of the codon-anticodon helix. Nucleic Acids Res..

[170] Kimsey I.J., Petzold K., Sathyamoorthy B., Stein Z.W., Al-Hashimi H.M. (2015). Visualizing transient Watson–Crick-like mispairs in DNA and RNA duplexes. Nature..

[171] Cantara W.A., Murphy F.V.t., Demirci H., Agris P.F. (2013). Expanded use of sense codons is regulated by modified cytidines in tRNA. Proc. Natl. Acad. Sci. USA.

[172] Grosjean H., Westhof E. (2016). An integrated, structure- and energy-based view of the genetic code. Nucleic Acids Res..

[173] Manickam N., Joshi K., Bhatt M.J., Farabaugh P.J. (2016). Effects of tRNA modification on translational accuracy depend on intrinsic codon-anticodon strength. Nucleic Acids Res..

[174] Vendeix F.A., Dziergowska A., Gustilo E.M., Graham W.D., Sproat B., Malkiewicz A., Agris P.F. (2008). Anticodon domain modifications contribute order to tRNA for ribosome-mediated codon binding. Biochemistry.

[175] Stuart J.W., Gdaniec Z., Guenther R., Marszalek M., Sochacka E., Malkiewicz A., Agris P.F. (2000). Functional anticodon architecture of human tRNA^Lys3^ includes disruption of intraloop hydrogen bonding by the naturally occurring amino acid modification, t^6^A. Biochemistry.

[176] Durant P.C., Bajji A.C., Sundaram M., Kumar R.K., Davis D.R. (2005). Structural effects of hypermodified nucleosides in the *Escherichia coli* and human tRNA^Lys^ anticodon loop: The effect of nucleosides s^2^U, mcm^5^U, mcm^5^s^2^U, mnm^5^s^2^U, t^6^A, and ms^2^t^6^A. Biochemistry.

[177] Witts R.N., Hopson E.C., Koballa D.E., Van Boening T.A., Hopkins N.H., Patterson E.V., Nagan M.C. (2013). Backbone-base interactions critical to quantum stabilization of transfer RNA anticodon structure. J. Phys. Chem. B.

[178] McCrate N.E., Varner M.E., Kim K.I., Nagan M.C. (2006). Molecular dynamics simulations of human tRNA^Lys,3UUU^: The role of modified bases in mRNA recognition. Nucleic Acids Res..

[179] Murphy F.V., Ramakrishnan V., Malkiewicz A., Agris P.F. (2004). The role of modifications in codon discrimination by tRNA^LysUUU^. Nat. Struct. Mol. Biol..

[180] Lamichhane T.N., Blewett N.H., Crawford A.K., Cherkasova V.A., Iben J.R., Begley T.J., Farabaugh P.J., Maraia R.J. (2013). Lack of tRNA modification isopentenyl-A37 alters mRNA decoding and causes metabolic deficiencies in fission yeast. Mol. Cell. Biol..

[181] Lamichhane T.N., Mattijssen S., Maraia R.J. (2013). Human cells have a limited set of tRNA anticodon loop substrates of the tRNA isopentenyltransferase TRIT1 tumor suppressor. Mol. Cell. Biol..

[182] Urbonavicius J. (2003). Transfer RNA modifications that alter +1 frameshifting in general fail to affect -1 frameshifting. RNA.

[183] Wei F.Y., Tomizawa K. (2012). Development of type 2 diabetes caused by a deficiency of a tRNA^lys^ modification. Islets.

[184] Takahashi N., Wei F.Y., Watanabe S., Hirayama M., Ohuchi Y., Fujimura A., Kaitsuka T., Ishii I., Sawa T., Nakayama H. (2017). Reactive sulfur species regulate tRNA methylthiolation and contribute to insulin secretion. Nucleic Acids Res..

[185] Waas W.F., Druzina Z., Hanan M., Schimmel P. (2007). Role of a tRNA base modification and its precursors in frameshifting in eukaryotes. J. Biol. Chem..

[186] De Crecy-Lagard V., Brochier-Armanet C., Urbonavicius J., Fernandez B., Phillips G., Lyons B., Noma A., Alvarez S., Droogmans L., Armengaud J. (2010). Biosynthesis of wyosine derivatives in tRNA: An ancient and highly diverse pathway in Archaea. Mol. Biol. Evol..

[187] Sample P.J., Koreny L., Paris Z., Gaston K.W., Rubio M.A., Fleming I.M., Hinger S., Horakova E., Limbach P.A., Lukes J. (2015). A common tRNA modification at an unusual location: The discovery of wyosine biosynthesis in mitochondria. Nucleic Acids Res..

[188] Smith C., Schmidt P.G., Petsch J., Agris P.F. (1985). Nuclear magnetic resonance signal assignments of purified [^13^C]methyl-enriched yeast phenylalanine transfer ribonucleic acid. Biochemistry.

[189] Matuszewski M., Sochacka E. (2014). Stability studies on the newly discovered cyclic form of tRNA *N*^6^-threonylcarbamoyladenosine (ct^6^A). Bioorg. Med. Chem. Lett..

[190] Miyauchi K., Kimura S., Suzuki T. (2013). A cyclic form of *N*^6^-threonylcarbamoyladenosine as a widely distributed tRNA hypermodification. Nat. Chem. Biol..

[191] Kang B.-i., Miyauchi K., Matuszewski M., D’Almeida G.S., Rubio M.A.T., Alfonzo J.D., Inoue K., Sakaguchi Y., Suzuki T., Sochacka E. (2017). Identification of 2-methylthio cyclic *N*^6^-threonylcarbamoyladenosine (ms^2^ct^6^A) as a novel RNA modification at position 37 of tRNAs. Nucleic Acids Res..

[192] Kim S.H., Sussman J.L., Suddath F.L., Quigley G.J., McPherson A., Wang A.H.J., Seeman N.C., Rich A. (1974). The general structure of transfer RNA molecules. Proc. Natl. Acad. Sci. USA.

[193] Ponnuswamy P.K., Gromiha M.M. (1994). On the conformational stability of oligonucleotide duplexes and tRNA molecules. J. Theor. Biol..

[194] Bjork G.R., Wikstrom P.M., Bystrom A.S. (1989). Prevention of translational frameshifting by the modified nucleoside 1-methylguanosine. Science.

[195] Gamper H.B., Masuda I., Frenkel-Morgenstern M., Hou Y.M. (2015). Maintenance of protein synthesis reading frame by EF-P and m^1^G37-tRNA. Nat. Commun..

[196] Brulé H., Holmes W.M., Keith G., Giegé R., Florentz C. (1998). Effect of a mutation in the anticodon of human mitochondrial tRNA^Pro^ on its post-transcriptional modification pattern. Nucleic Acids Res..

[197] Swinehart W.E., Jackman J.E. (2015). Diversity in mechanism and function of tRNA methyltransferases. RNA Biol..

[198] Brulé H., Elliott M., Redlak M., Zehner Z.E., Holmes W.M. (2004). Isolation and characterization of the human tRNA-(N^1^G37) methyltransferase (TRM5) and comparison to the *Escherichia coli* TrmD protein. Biochemistry.

[199] Powell C.A., Kopajtich R., D’Souza A.R., Rorbach J., Kremer L.S., Husain R.A., Dallabona C., Donnini C., Alston C.L., Griffin H. (2015). TRMT5 mutations cause a defect in post-transcriptional modification of mitochondrial tRNA associated with multiple respiratory-chain deficiencies. Am. J. Hum. Genet..

[200] Yakovchuk P., Protozanova E., Frank-Kamenetskii M.D. (2006). Base-stacking and base-pairing contributions into thermal stability of the DNA double helix. Nucleic Acids Res..

[201] Butcher S.E., Pyle A.M. (2011). The molecular interactions that stabilize RNA tertiary structure: RNA motifs, patterns, and networks. Acc. Chem. Res..

[202] Bujalowski W., Graeser E., McLaughlin L.W., Porschke D. (1986). Anticodon loop of tRNA^Phe^: Structure, dynamics, and Mg^2+^ binding. Biochemistry.

[203] Wells B.D. (1984). The conformation of the tRNA^Phe^ anticodon loop monitored by fluorescence. Nucleic Acids Res..

[204] Wolfson J.M., Kearns D.R. (1975). Europium as a fluorescent probe of transfer RNA structure. Biochemistry.

[205] Chen Y., Sierzputowska-Gracz H., Guenther R., Everett K., Agris P.F. (1993). 5-methylcytidine is required for cooperative binding of Mg^2+^ and a conformational transition at the anticodon stem-loop of yeast phenylalanine tRNA. Biochemistry.

[206] Dao V., Guenther R.H., Agris P.F. (1992). The role of 5-methylcytidine in the anticodon arm of yeast tRNA^Phe^: site-specific Mg^2+^ binding and coupled conformational transition in DNA analogs. Biochemistry.

[207] Yue D., Kintanar A., Horowitz J. (1994). Nucleoside modifications stabilize Mg^2+^ binding in *Escherichia coli* tRNA^Val^: An imino proton NMR investigation. Biochemistry.

[208] Maglott E.J., Deo S.S., Przykorska A., Glick G.D. (1998). Conformational transitions of an unmodified tRNA: Implications for RNA folding. Biochemistry.

[209] Jones C., Spencer A.C., Hsu J.L., Spremulli L., Martinis S.A., DeRider M., Agris P.F. (2006). A counterintuitive Mg^2+^-dependent and modification-assisted functional folding of mitochondrial tRNAs. J. Mol. Biol..

[210] Schweizer M.P., De N., Pulsipher M., Brown M., Reddy P.R., Petrie C.R., Chheda G.B. (1984). Quantitative aspects of metal ion binding to certain transfer RNA anticodon loop modified nucleosides. Biochim. Biophys. Acta.

[211] Varnagy K., Jezowska-Bojczuk M., Swiatek J., Kozlowski H., Sovago I., Adamiak R.W. (1990). Metal binding ability of hypermodified nucleosides of t-RNA. Potentiometric and spectroscopic studies on the metal complexes of *N*-[(9-β-d-ribofuranosylpurin-6-yl)-carbamoyl] threonine. J. Inorg. Biochem..

[212] Cabello-Villegas J., Tworowska I., Nikonowicz E.P. (2004). Metal ion stabilization of the U-turn of the A_37_
*N*^6^-dimethylallyl-modified anticodon stem-loop of *Escherichia coli* tRNA^Phe^. Biochemistry.

[213] Auffinger P., Westhof E. (1999). Singly and bifurcated hydrogen-bonded base-pairs in tRNA anticodon hairpins and ribozymes. J. Mol. Biol..

[214] Olejniczak M., Uhlenbeck O.C. (2006). tRNA residues that have coevolved with their anticodon to ensure uniform and accurate codon recognition. Biochimie.

[215] Baldi M.I., Mattoccia E., Bufardeci E., Fabbri S., Tocchini-Valentini G.P. (1992). Participation of the intron in the reaction catalyzed by the *Xenopus* tRNA splicing endonuclease. Science.

[216] Ogle J.M., Brodersen D.E., Clemons W.M., Tarry M.J., Carter A.P., Ramakrishnan V. (2001). Recognition of cognate transfer RNA by the 30S ribosomal subunit. Science.

[217] Bullock T.L., Uter N., Nissan T.A., Perona J.J. (2003). Amino acid discrimination by a class I aminoacyl-tRNA synthetase specified by negative determinants. J. Mol. Biol..

[218] Yaremchuk A., Tukalo M., Grotli M., Cusack S. (2001). A succession of substrate induced conformational changes ensures the amino acid specificity of *Thermus thermophilus* prolyl-tRNA synthetase: Comparison with histidyl-tRNA synthetase. J. Mol. Biol..

[219] Cantara W.A., Bilbille Y., Kim J., Kaiser R., Leszczynska G., Malkiewicz A., Agris P.F. (2012). Modifications modulate anticodon loop dynamics and codon recognition of *E. coli* tRNA^Arg1,2^. J. Mol. Biol..

[220] Vangaveti S., Ranganathan S.V. (2017). Personal communication.

[221] Ashraf S.S., Ansari G., Guenther R., Sochacka E., Malkiewicz A., Agris P.F. (1999). The uridine in “U-turn”: Contributions to tRNA-ribosomal binding. RNA.

[222] von Ahsen U., Green R., Schroeder R., Noller H.F. (1997). Identification of 2’-hydroxyl groups required for interaction of a tRNA anticodon stem-loop region with the ribosome. RNA.

[223] Phelps S.S., Jerinic O., Joseph S. (2002). Universally conserved interactions between the ribosome and the anticodon stem-loop of A site tRNA important for translocation. Mol. Cell.

[224] Sundaram M., Durant P.C., Davis D.R. (2000). Hypermodified nucleosides in the anticodon of tRNA^Lys^ stabilize a canonical U-turn structure. Biochemistry.

[225] Phizicky E.M., Hopper A.K. (2010). tRNA biology charges to the front. Genes Dev..

[226] Gerber A.P., Keller W. (1999). An adenosine deaminase that generates inosine at the wobble position of tRNAs. Science.

[227] Björk G.R., Jacobsson K., Nilsson K., Johansson M.J., Bystrom A.S., Persson O.P. (2001). A primordial tRNA modification required for the evolution of life?. EMBO J..

[228] El Yacoubi B., Lyons B., Cruz Y., Reddy R., Nordin B., Agnelli F., Williamson J.R., Schimmel P., Swairjo M.A., de Crecy-Lagard V. (2009). The universal YrdC/Sua5 family is required for the formation of threonylcarbamoyladenosine in tRNA. Nucleic Acids Res..

[229] Durant P.C., Davis D.R. (1999). Stabilization of the anticodon stem-loop of tRNA^Lys,3^ by an A+-C base-pair and by pseudouridine. J. Mol. Biol..

[230] Newby M.I., Greenbaum N.L. (2001). A conserved pseudouridine modification in eukaryotic U2 snRNA induces a change in branch-site architecture. RNA.

[231] Astrom S.U., Bystrom A.S. (1994). Rit1, a tRNA backbone-modifying enzyme that mediates initiator and elongator tRNA discrimination. Cell.

[232] Demeshkina N., Jenner L., Westhof E., Yusupov M., Yusupova G. (2012). A new understanding of the decoding principle on the ribosome. Nature.

[233] Parker J. (1989). Errors and alternatives in reading the universal genetic code. Microbiol. Rev..

[234] Roth A.C. (2012). Decoding properties of tRNA leave a detectable signal in codon usage bias. Bioinformatics.

[235] Hopper A.K. (2013). Transfer RNA post-transcriptional processing, turnover, and subcellular dynamics in the yeast *Saccharomyces cerevisiae*. Genetics.

[236] Chawla M., Oliva R., Bujnicki J.M., Cavallo L. (2015). An atlas of RNA base pairs involving modified nucleobases with optimal geometries and accurate energies. Nucleic Acids Res..

[237] Vendeix F.A.P., Munoz A.M., Agris P.F. (2009). Free energy calculation of modified base-pair formation in explicit solvent: A predictive model. RNA.

