# Supplementary material for: Chemical and Conformational Diversity of Modified Nucleosides Affects tRNA Structure and Function
Source: Biomolecules. 2017 Mar 16;7(1):29. doi: 10.3390/biom7010029 (PMC5372741; doi:10.3390/biom7010029)
Supplement: Supplementary File 1 [file biomolecules-07-00029-s001.pdf]

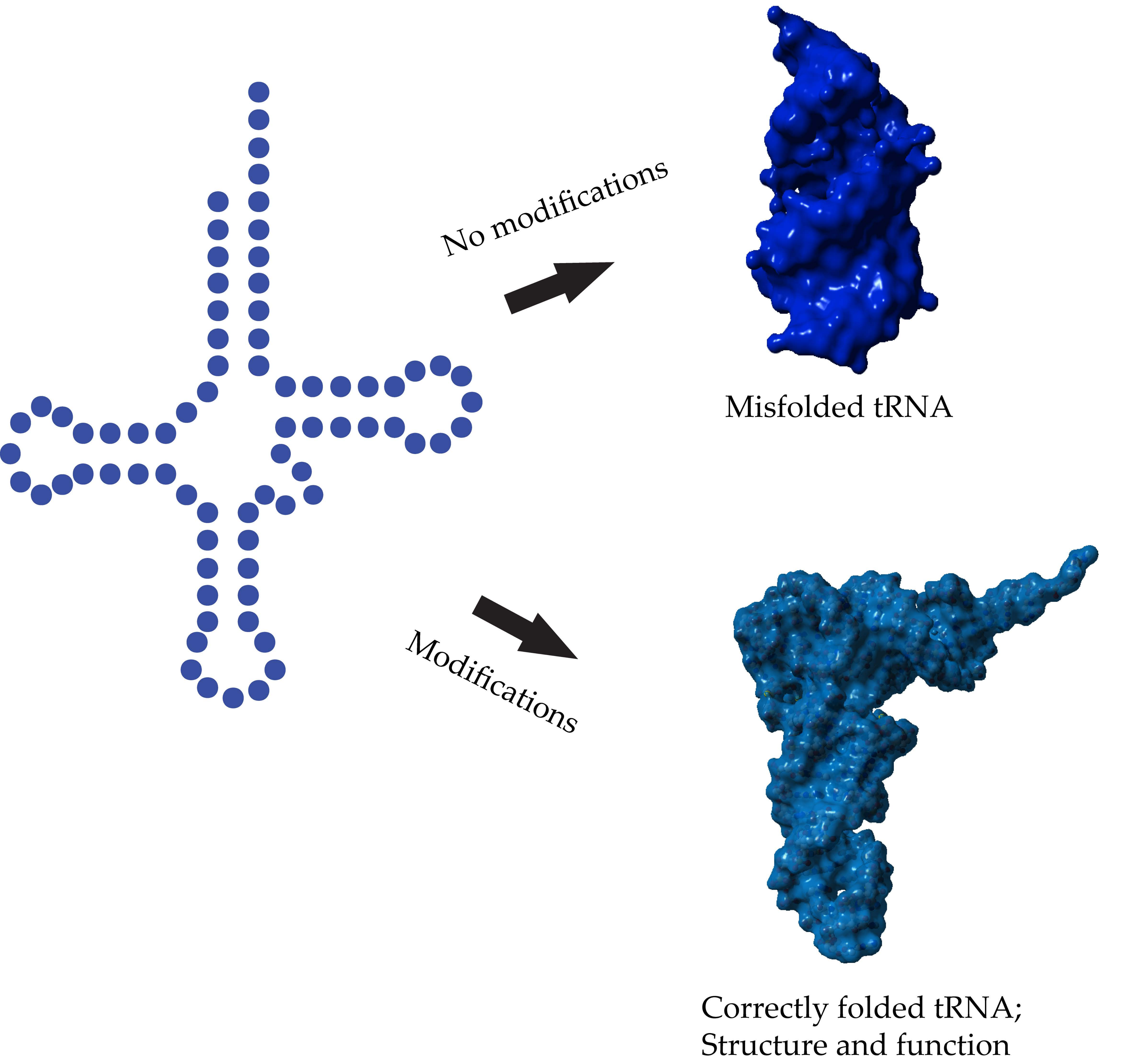
No modifications
Misfolded tRNA
Modifications
Correctly folded tRNA;
Structure and function
